# Supramolecular Aptamers on Graphene Oxide for Efficient Inhibition of Thrombin Activity

**DOI:** 10.3389/fchem.2019.00280

**Published:** 2019-05-16

**Authors:** Ting-Xuan Lin, Pei-Xin Lai, Ju-Yi Mao, Han-Wei Chu, Binesh Unnikrishnan, Anisha Anand, Chih-Ching Huang

**Affiliations:** ^1^Department of Bioscience and Biotechnology, National Taiwan Ocean University, Keelung, Taiwan; ^2^Doctoral Degree Program in Marine Biotechnology, National Taiwan Ocean University, Keelung, Taiwan; ^3^Doctoral Degree Program in Marine Biotechnology, Academia Sinica, Taipei, Taiwan; ^4^Center of Excellence for the Oceans, National Taiwan Ocean University, Keelung, Taiwan; ^5^School of Pharmacy, College of Pharmacy, Kaohsiung Medical University, Kaohsiung, Taiwan

**Keywords:** thrombin, aptamer, graphene oxide, self-assembly, anticoagulation

## Abstract

Graphene oxide (GO), a two-dimensional material with a high aspect ratio and polar functional groups, can physically adsorb single-strand DNA through different types of interactions, such as hydrogen bonding and π-π stacking, making it an attractive nanocarrier for nucleic acids. In this work, we demonstrate a strategy to target exosites I and II of thrombin simultaneously by using programmed hybrid-aptamers for enhanced anticoagulation efficiency and stability. The targeting ligand is denoted as Supra-TBA_15/29_ (supramolecular TBA_15/29_), containing TBA_15_ (a 15-base nucleotide, targeting exosite I of thrombin) and TBA_29_ (a 29-base nucleotide, targeting exosite II of thrombin), and it is designed to allow consecutive hybridization of TBA_15_ and TBA_29_ to form a network of TBAs (i.e., supra-TBA_15/29_). The programmed hybrid-aptamers (Supra-TBA_15/29_) were self-assembled on GO to further boost anticoagulation activity by inhibiting thrombin activity, and thus suppress the thrombin-induced fibrin formation from fibrinogen. The Supra-TBA_15/29_-GO composite was formed mainly through multivalent interaction between poly(adenine) from Supra-TBA_15/29_ and GO. We controlled the assembly of Supra-TBA_15/29_ on GO by regulating the preparation temperature and the concentration ratio of Supra-TBA_15/29_ to GO to optimize the distance between TBA_15_ and TBA_29_ units, aptamer density, and aptamer orientation on the GO surfaces. The dose-dependent thrombin clotting time (TCT) delay caused by Supra-TBA_15/29_-GO was >10 times longer than that of common anticoagulant drugs including heparin, argatroban, hirudin, and warfarin. Supra-TBA_15/29_-GO exhibits high biocompatibility, which has been proved by *in vitro* cytotoxicity and hemolysis assays. In addition, the thromboelastography of whole-blood coagulation and rat-tail bleeding assays indicate the anticoagulation ability of Supra-TBA_15/29_-GO is superior to the most widely used anticoagulant (heparin). Our highly biocompatible Supra-TBA_15/29_-GO with strong multivalent interaction with thrombin [dissociation constant (*K*_d_) = 1.9 × 10^−11^ M] shows great potential as an effective direct thrombin inhibitor for the treatment of hemostatic disorders.

**Graphical Abstract:**
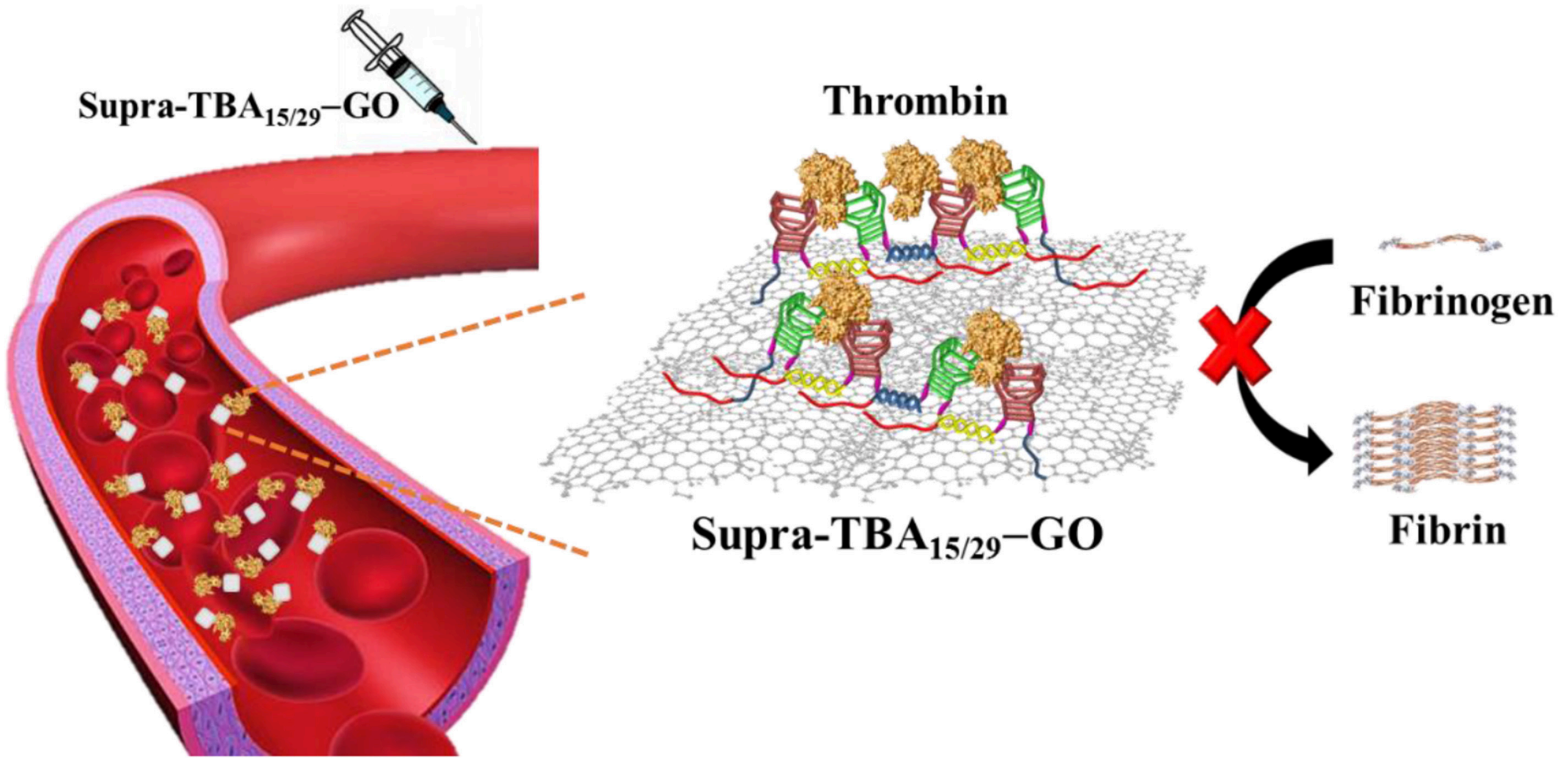
A programmed thrombin binding aptamers immobilized on graphene oxide for strong inhibition of thrombin activity by targeting the exosites I and II of thrombin simultaneously.

## Introduction

Stable and uninterrupted blood flow is important for maintaining a healthy life style. The human body has highly efficient bi-directional regulation of coagulation and hemolysis to prevent excessive blood flow out of injured wounds by forming clots and breaking down the clot when not needed (Doolittle, 2016; Rana and Neeves, 2016). In general, artificial triggering of blood coagulation processes is not needed, except in congenital hemophilia patients who lack factor VIII or factor IX, which can be treated by blood transfusion or injection of clotting factor (Morfini et al., 2013). On the contrary, many diseases are caused by the formation of thrombus (Previtali et al., 2011; Otsuka et al., 2016). Currently, predicting the exact place of thrombus formation in the body is difficult. So, when symptoms arise, serious issues tend to follow, which cause hemorrhage and ischemic necrosis of tissue/organs (Previtali et al., 2011; Suppiej et al., 2015). Therefore, preventing or controlling the clot formation in such cases is necessary. In a normal coagulation system, complex interactions of coagulation factors, platelets, cofactors, and regulators maintain homeostasis for the systematic regulation of bleeding and thrombus formation (Palta et al., 2014). The coagulation reaction has three pathways including the intrinsic pathway, the extrinsic pathway, and the common pathway (Gailani and Renné, 2007; Mackman et al., 2007). Among all the coagulation factors, thrombin (activated Factor II)—a glycosylated serine protease—is indispensable in the coagulation mechanism (Crawley et al., 2007; Smith et al., 2015). Its main function is to take charge of the most crucial and last step in the coagulation cascade reaction; the cleavage of fibrinogen and its activation into fibrin. Only then can fibrin bind to the glycoprotein IIb/IIIa receptor on platelets and connect with platelets and other coagulation factors to form a more solid clot in order to achieve hemostasis (Crawley et al., 2007). Therefore, targeting thrombin activity could be an effective strategy to control thrombosis. Heparin, argatroban, hirudin, and dabigatran are the commonly used anticoagulant drugs which inhibit thrombin activity (Lee and Ansell, 2011; Alquwaizani et al., 2013). Heparin is one of the common anticoagulants in clinical treatment of pulmonary embolism, venous thrombosis, and cerebral embolism (Jin et al., 1997; Zhang et al., 2018). However, heparin may cause an immune-mediated coagulation side effect of heparin-induced thrombocytopenia (HIT) (Pollak et al., 2011). HIT causes abnormal coagulation of platelets and triggers thrombosis symptoms, and the incidence is higher for high-molecular-weight heparin than that of low-molecular-weight heparin. Therefore, developing an anticoagulant with high stability and low side effects is an important and challenging issue.

Aptamers are short DNA or RNA strands selected *in vitro* or *in vivo* through systematic evolution of ligands by exponential enrichment (SELEX) for strong and specific recognition of their targets (Darmostuk et al., 2015; Huang et al., 2016a; Lyu et al., 2016; Pang et al., 2018). In the past two decades, many aptamers have been selected to target different anticoagulation factors for anticoagulation applications (Pagano et al., 2008; Nimjee et al., 2016; Zavyalova et al., 2016a; Chabata et al., 2018). However, many of them suffer from poor specificity, weak binding strength and can be easily degraded by nucleases present in the blood, which limit their successful applications *in vivo*. Even so, a 15-mer thrombin-binding aptamer (TBA_15_) with specific G-quadruplex structures, which can resist digestion by nucleases, has been shown to extend anticoagulation activity in whole blood (Bock et al., 1992). TBA_15_ binds with thrombin (dissociation constant (*K*_d_) of ~100 nM) at the fibrinogen-binding exosite I, resulting in the inhibition of thrombin activity (Padmanabhan et al., 1993). However, very high concentrations of TBA_15_ (in micromolar regime) are required to achieve an appropriate anticoagulant response due to its low binding affinity. Another 29-mer thrombin-binding aptamer (TBA_29_) exhibits much stronger binding with exosite II of thrombin (*K*_d_ ~ 0.5 nM) (Tasset et al., 1997); however, it could not inhibit thrombin activity toward the formation of fibrin from fibrinogen and thrombin-mediated platelet activation. Moreover, the short half-life of blood circulation of TBAs diminishes their anticoagulation potency *in vivo*.

Compared to free aptamers, aptamer-conjugated nanoparticles provide ultrahigh local aptamer densities on nanoparticle surfaces to increase their binding affinity to thrombin and resistance toward nuclease digestion (Liu et al., 2014; Jo and Ban, 2016; Urmann et al., 2016; Yang et al., 2018). In addition, each nanoparticle can be conjugated with different functional aptamers for multivalent binding of target proteins. A number of TBA-based nanocomposites have been developed by modification of TBAs on metallic, polymeric, and DNA origami nanoparticles for efficient binding of TBAs with thrombin to increase their anticoagulation potency (Rinker et al., 2008; Kim et al., 2010; Musumeci and Montesarchio, 2012; Riccardi et al., 2017; Kumar and Seminario, 2018; Lai et al., 2018). However, anchoring of TBAs on the nanoparticles requires extensive and tedious functionalization processes. In addition, the steric position, distance, and orientation of TBAs on the nanoparticles are difficult to manipulate. Thus, these nanocomposites are rarely employed for anticoagulation *in vivo* (Lai et al., 2018).

**Scheme 1:**
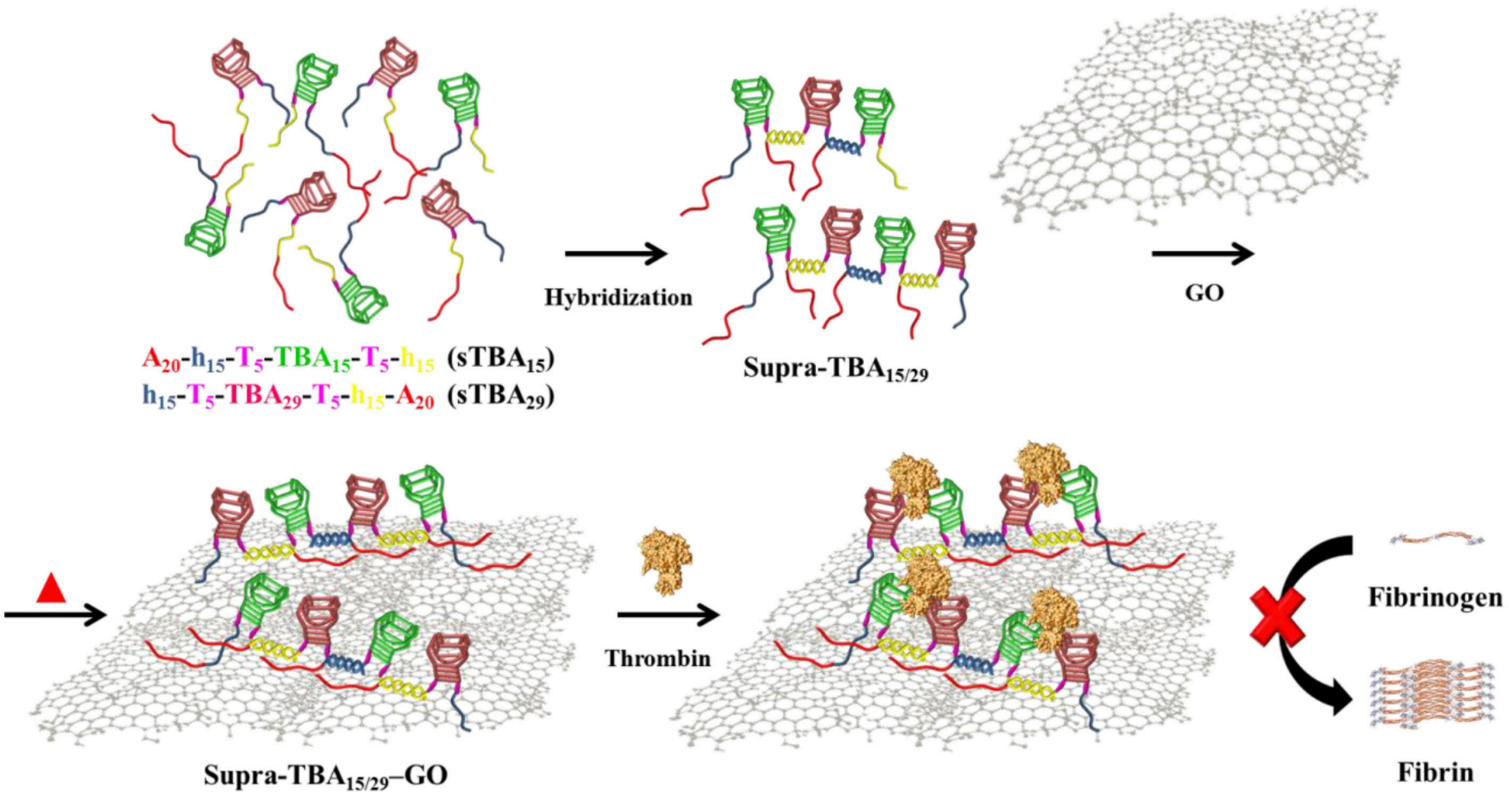
Schematic representation of the synthesis of self-assembled programmed hybrid thrombin-binding aptamers on graphene oxide and their multivalent interaction with thrombin for enhanced anticoagulant activity.

Graphene oxide (GO) has been reported to adsorb single-stranded nucleic acid chains by cooperative van der Waals' force, π-π stacking interaction, and hydrogen bonding interaction (Antony and Grimme, 2008; Varghese et al., 2009; Wu et al., 2011; Chen et al., 2014; Liu et al., 2016). Recently, some aptamer-adsorbed GO have been demonstrated for protein detection and cell labeling (Pu et al., 2011; Gao et al., 2015; Kim et al., 2015; Xiao et al., 2017). Most aptamers adopt specific conformational structures, which enable high specificity toward the targeting molecule (Rowsell et al., 1998; Tucker et al., 2012; Zavyalova et al., 2016b,c; Krauss et al., 2018). However, these unique conformational structures of aptamers are usually disrupted after adsorption onto GO. Moreover, the aptamer-adsorbed GO are not stable in human plasma due to the competitive adsorption between plasma components, such as high concentration proteins and aptamers on GO. As a result, the adsorbed aptamers tend to desorb from GO in human plasma (Wang et al., 2010; Mao et al., 2015; Zhu et al., 2015; Lu et al., 2016). In this study, we developed a simple strategy to target exosites I and II of thrombin simultaneously by using programmed hybrid-TBAs immobilized on partially reduced GO for enhanced stability of the TBAs and improved anticoagulation efficiency (Scheme 1). The targeting ligand is denoted as Supra-TBA_15/29_ [–(A_20_h_15_T_5_TBA_15/29_T_5_h_15_)_*n*_–], containing a supramolecular structure of consecutive hybrid TBA_15_/TBA_29_ units with multiple poly(adenine) (A_20_) segments for immobilization on GO. GO has been shown to preferentially interact with single stranded (ss) DNA with poly(adenine) sequences (Ranganathan et al., 2016). The multi-segment A_20_ segments allow Supra-TBA_15/29_ to anchor strongly on the GO surface, which results in the high stability of Supra-TBA_15/29_-GO nanocomposites in human plasma. The Supra-TBA_15/29_-GO nanocomposites possess superior anticoagulant activity to free both TBAs (TBA_15_ or TBA_29_) and Supra-TBA_15/29_. In addition, thromboelastography of whole-blood coagulation and rat-tail bleeding assays further demonstrate the strong anticoagulation ability of Supra-TBA_15/29_-GO.

## Results and Discussion

### Formation of Supra-TBA_15/29_

The oligonucleotide sequences of A_20_h_15_T_5_TBA_15_T_5_h_15_ (sTBA_15_) and h_15_T_5_TBA_29_T_5_h_15_A_20_ (sTBA_29_) are listed in Table S1 (Supporting Information). The sTBA_15_ and sTBA_29_ comprised of three blocks, a 20-polyadenine (A_20_) for anchoring on GO, two 15-base sequences (h_15_) for consecutive hybridization, a 5-repeat thymidine (T_5_) as a linker, and a TBA sequence providing functionality. TBA_29_ has a G-quadruplex structure with a loop and stem in the terminal, while TBA_15_ has only a G-quadruplex structure (Macaya et al., 1993; Padmanabhan et al., 1993). A previous study indicated that having two 7-mers inserted on each side of TBA_15_ unit introduces a loop and stem structure for stabilizing the G-quadruplex structures, thereby increasing their inhibitory potency (Hsu et al., 2012). By simply mixing sTBA_15_ and sTBA_29_ in phosphate-buffered saline (PBS; containing 25.0 mM tris-HCl, 150.0 mM NaCl, 5 mM KCl, 1.0 mM MgCl_2_, and 1.0 M CaCl_2_; adjusted to pH 7.4 using HCl) solution, the Supra-TBA_15/29_ were formed through the consecutive hybridization of the h_15_ sequences (Scheme S1A, Supporting Information). In addition, we also prepared dimer TBA_15_/TBA_29_ (di-TBA_15/29_) by mixing sTBA_15_ and dTBA_29_ (nh_15_T_5_TBA_29_T_5_h_15_A_20_). The di-TBA_15/29_ are formed by a single step hybridization between sTBA_15_ and dTBA_29_ (Scheme S1B). A control experiment with a mixture of sTBA_15_ and nTBA_29_ (nh_15_T_5_TBA_29_T_5_nh_15_A_20_) indicated that they could not hybridize with each other (Scheme S1C). For simplicity, we denote the mixture of sTBA_15_ and nTBA_29_ as nh-TBA_15/29_. The hydrodynamic size of Supra-TBA_15/29_ (~207.8 nm) was larger than sTBA_15_ (~24.3 nm), sTBA_29_ (~25.6 nm), di-TBA_15/29_ (~53.7 nm), and the mixture of sTBA_15_ and nTBA_29_ (~26.3 nm), determined by dynamic light scattering (DLS), which suggested the formation of a supramolecular TBA structure. The lower electrophoretic mobility of Supra-TBA_15/29_ compared to that of sTBA_15_, sTBA_29_, and di-TBA_15/29_ further confirmed the formation of Supra-TBA_15/29_ through hybridization (Figure S1, Supporting Information) (Hellman and Fried, 2007). The gel electrophoresis result of Supra-TBA_15/29_ shows a broad band, probably due to the formation of varying lengths of Supra-TBA_15/29_. Based on the location and the width of the broad band, we propose that our Supra-TBA_15/29_ consists of mainly 2–6 hybridized TBA units.

### Thrombin Clotting Time of Supra-TBA_15/29_

**Figure 1:**
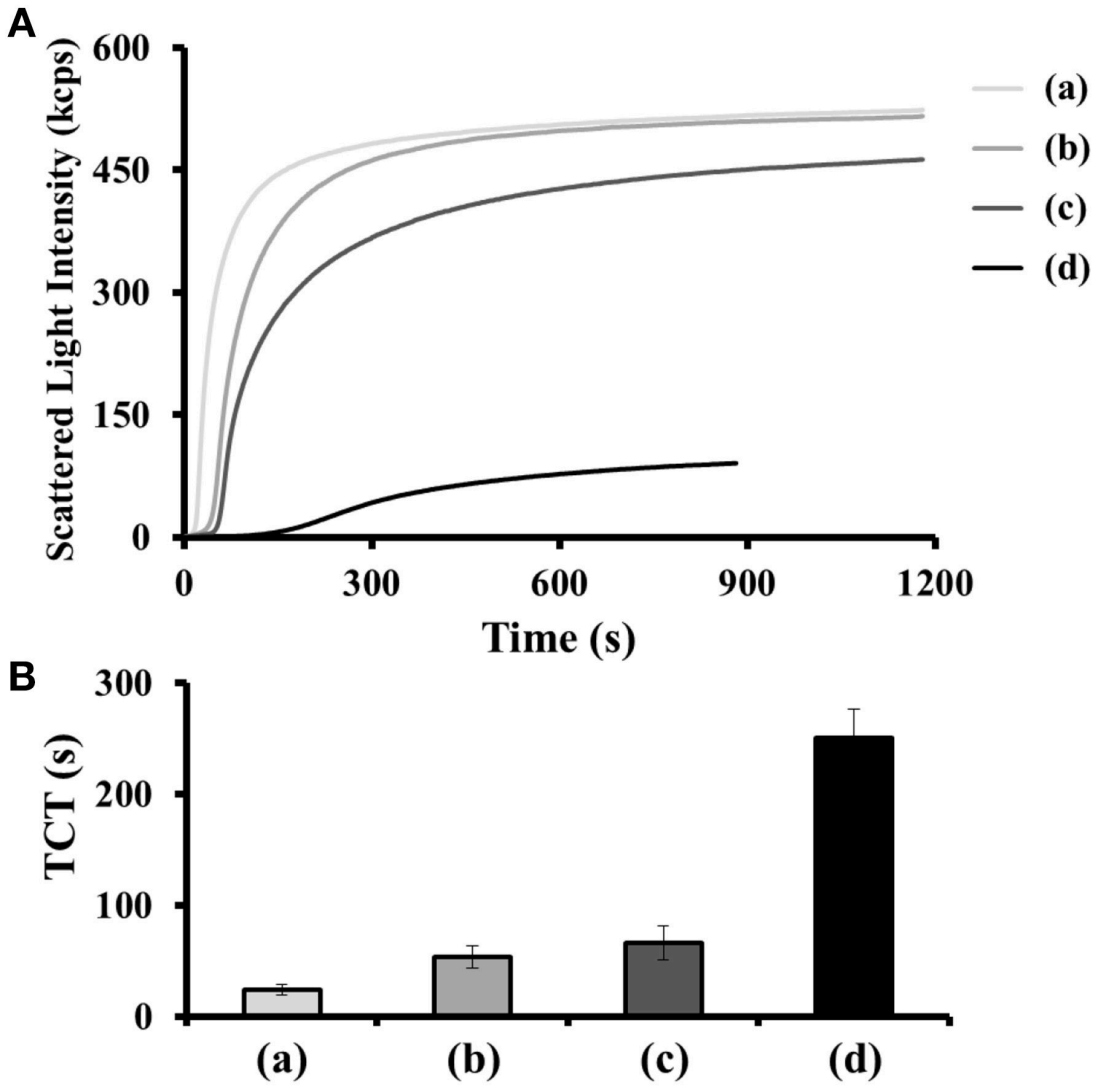
**(A)** Scattering light intensity as a function of time for coagulating mixtures of thrombin (15 nM), human plasma (2-fold diluted), BSA (100 μM) in the **(a)** absence and **(b–d)** presence of **(b)** nh-TBA_15/29_, **(c)** di-TBA_15/29_, and **(d)** Supra-TBA_15/29_ with a concentration of 100 nM (in terms of TBA). **(B)** TCT obtained from the light scattering intensities generated by the coagulation process. The TCT for each sample was noted as the time at which the differential scattering signal intensity reached the maximum. The longest time monitored in the clotting assays was set at 1,200 s. Error bars represent the standard deviations of experiments in triplicate.

We evaluated the inhibitory ability of Supra-TBA_15/29_ against thrombin in human plasma by thrombin clotting time (TCT) assays. The TCT test is a reliable diagnostic tool for bleeding and/or clotting disorder and screening of coagulation factors I (fibrinogen), IIa (thrombin), and XIII (fibrillation stabilizing factor) in the common coagulation pathways (Ignjatovic, 2013). We investigated the thrombin inhibitory activity of nh-TBA_15/29_, di-TBA_15/29_, and Supra-TBA_15/29_, which were prepared by mixing sTBA_15_ with nTBA_29_, dTBA_29_, and sTBA_29_ (Scheme S1), respectively. We compared the inhibitory potencies of nh-TBA_15/29_, di-TBA_15/29_, and Supra-TBA_15/29_ by real-time kinetics of coagulation, through recording the scattering light intensity as a function of time (Figure 1A). Fibrinogen (factor I) is a fibrous glycoprotein (~45 nm) with a complex structure. It consists of three pairs of polypeptide chains (α-, β-, and γ-chains) linked together by 29 disulfide bonds (Mosesson, 2005). Soluble fibrinogen is converted into protofibrils by the cleavage of fibrinopeptides Aα and Bβ in the central region by thrombin via intermolecular interactions of knobs “A” and “B” in the central nodule and holes “a” and “b” at the ends of the molecules. The protofibrils further aggregate laterally to form fibers and then branch to form a three-dimensional network of the fibrin clots. The higher activity of thrombin results in the formation of a larger fibrin gel, which causes increased scattering of light. The concentration of the total of the TBAs was held constant (100 nM) in these experiments. Compared with the control (no inhibitor), which has a TCT value of 24 ± 1 s, Supra-TBA_15/29_ prolonged the clotting time to 251 ± 21 s. The TCT values for nh-TBA_15/29_ and di-TBA_15/29_ were 54 ± 10 and 66 ± 15 s, respectively. The inhibitory activity of Supra-TBA_15/29_ was superior to nh-TBA_15/29_ and di-TBA_15/29_ mainly due to synergistic effect. That is, simultaneous binding and blocking by the two aptamers of both of the exosites (exosite I and II) of thrombin and steric hindrance of relatively large Supra-TBA_15/29_ to fibrinogen from accessing thrombin led to strong and synergistic inhibition of the thrombin-dependent coagulant activity (Hsu et al., 2011).

### Characterization of GO and Supra-TBA_15/29_-GO

GO was synthesized by improved Hummers' method from graphite powder with a particle size of 7–11 μm (Hummers and Offeman, 1958; Marcano et al., 2010). The detailed procedure of synthesis of GO is given in the experimental section. The TEM image in Figure S2A (Supporting Information) shows that most of the as-synthesized GO with size ca. 200–300 nm are single layered. Atomic force microscopy (AFM) showed that the average size of a single-layer GO was ~230 nm, and the monolayer thickness was about 1.1 nm (Figure S2B, Supporting Information). We prepared a series of Supra-TBA_15/29_-GO nanocomposites and studied their inhibitory activities against thrombin. The Supra-TBA_15/29_ ([TBA] = 2.5 μM) was mixed with GO (20–80 μg mL^−1^) in PBS containing 1.0 M NaCl at 25–90°C. After incubation for 2 h, the Supra-TBA_15/29_-GO solutions were purified through three centrifugation and washing cycles. The Supra-TBA_15/29_ on the GO was determined through the quantitation of unbound TBAs in the supernatant, and the results are listed in Table S2. The Supra-TBA_15/29_ modified on GO formed Supra-TBA_15/29_-GO mainly through van der Waals' force, π-π stacking, and hydrogen-bonding between poly(adenine) (A_20_) from Supra-TBA_15/29_ and GO (Antony and Grimme, 2008; Varghese et al., 2009; Chen et al., 2014). Previous studies revealed that purine bases (A and G) bind more strongly than the pyrimidines (T and C) (Park et al., 2014; Liu et al., 2016; Ranganathan et al., 2016). The DNA oligonucleotides possess much stronger [1–3 order(s) higher] association constants compared to the single nucleosides and longer DNA provides more binding sites for adsorption on GO. The AFM image of Supra-TBA_15/29_-GO shows that Supra-TBA_15/29_ was anchored on the surface of GO with a thickness of ~16 nm (Figure S2C). The AFM result suggests a monolayer of Supra-TBA_15/29_ was immobilized on either side of the GO, which is evident from the size of TBA (~2.0 nm) and the length of h_15_T_5_ (~6.8 nm; 0.34 nm bp^−1^).

Temperature may affect the structure and flexibility of Supra-TBA_15/29_ and is known to affect oxygen moieties on GO, which in turn could affect their interactions during Supra-TBA_15/29_-GO synthesis (Lin et al., 2010; Geggier et al., 2011; Brunet et al., 2018). Heat treatment altered the density of Supra-TBA_15/29_ on GO very significantly (Table S2) as a result of unfavorable entropy, partial denaturation of Supra-TBA_15/29_, and mild reduction of GO at higher temperatures. The adsorption density of the Supra-TBA_15/29_ on GO decreased with increasing concentration of GO used during the preparation of Supra-TBA_15/29_-GO conjugates. In the series of Supra-TBA_15/29_-GO prepared, the maximum adsorption density of Supra-TBA_15/29_ on GO (using 20 μg mL^−1^ of GO and at 25°C for the preparation of Supra-TBA_15/29_-GO) was calculated to be 47.1 nmol mg^−1^, which revealed a high density of Supra-TBA_15/29_ on the GO surface compared to the reported result for the saturated adsorption of poly-adenine (A_15_) on GO (~7.18 nmol mg^−1^) (Lu et al., 2016). We conducted UV-vis absorption spectroscopy (Figure S3), Fourier-transform infrared spectroscopy (FT-IR; Figure S4), X-ray photoelectron spectroscopy (XPS; Figure S5), Raman spectroscopy (Figure S6), and elemental analysis (EA; Table S2) to characterize GO and understand the effect of temperature on the degree of reduction of GO. Our results indicate GO underwent only a slight reduction even after treatment at 90°C for 2 h. However, this slight reduction of GO played a crucial role in the anticoagulation potency and stability of Supra-TBA_15/29_-GO in human plasma, which will be discussed in the following sections. The material is stored at 4°C when not in use and is stable up to 3 months.

### Anticoagulant Activity of Supra-TBA_15/29_-GO

**Figure 2:**
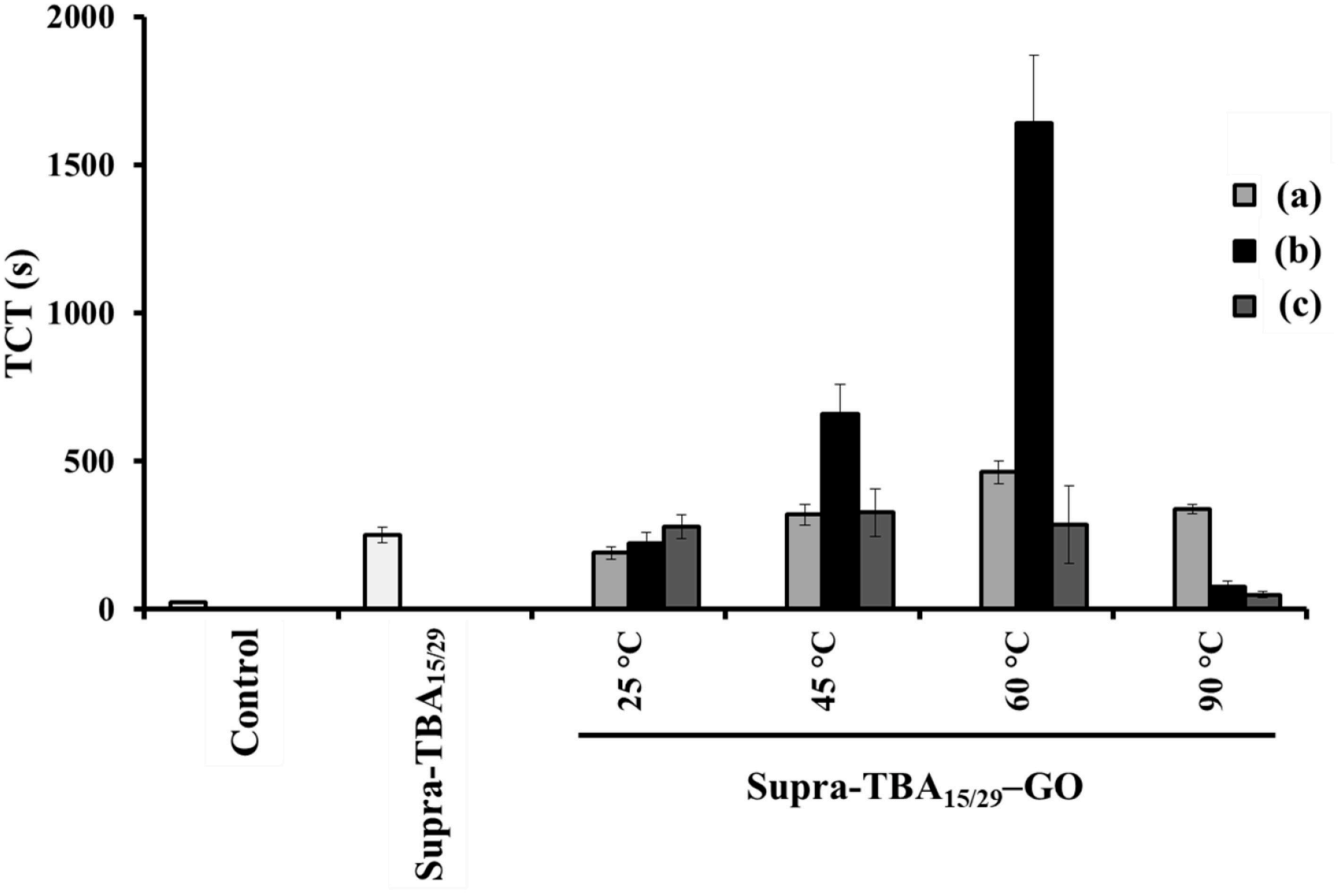
Thrombin clotting time (TCT) of Supra-TBA_15/29_ and Supra-TBA_15/29_-GO prepared at different concentrations of GO **(a)** 20, **(b)** 40, and **(c)** 80 μg mL^−1^ at different temperatures. The concentration of Supra-TBA_15/29_ in all samples was 100 nM. The TCT assay in the absence of inhibitors serves as a control group. The error bars represent the standard deviations of experiments in triplicate. Other conditions were the same as those described in Figure 1.

In contrast to GO, which tends to aggregate in PBS of high ionic strength, the Supra-TBA_15/29_-GO dispersion was very stable (no aggregation) when incubated in 1X PBS (Figure S7, Supporting Information). The hydrodynamic size of GO aggregates in PBS was ~1,800 nm, whereas the size of Supra-TBA_15/29_-GO (~150 nm) did not change significantly with increasing the concentration of PBS. The high density of TBAs with high negative charge mainly contributed to the high stability of Supra-TBA_15/29_-GO. On the other hand, the circular dichroism (CD) spectra of Supra-TBA_15/29_-GO prepared at different temperatures (25–90°C) did not show significant difference with that of Supra-TBA_15/29_ (Figure S8, Supporting Information), revealing that the G-quadruplex structure of TBAs were highly preserved after Supra-TBA_15/29_ were adsorbed on GO. The TCT of Supra-TBA_15/29_ and Supra-TBA_15/29_-GO prepared with various concentrations of GO (20, 40 and 80 μg mL^−1^) at 25, 45, 60, and 90°C is presented in Figure 2. TCT values for Supra-TBA_15/29_-GO prepared with GO (40 μg mL^−1^) at 25, 45, 60, and 90°C were ~9, 26, 66, and three times longer, respectively, than that which was in the absence of inhibitor. The higher inhibitory activity of Supra-TBA_15/29_-GO can be ascribed to the high specificity of the aptamers to block the exosite I and II binding sites and/or the active sites of thrombin for fibrinogen, and high ligand density on the GO surface. In addition, the 5′- and 3′-extended TBA molecules were anchored on GO through interactions between poly(adenine) and GO, which facilitated the exposure of major binding loops of TBA_15_ (T^3^T ^4^ and T^12^T^13^ loops) and TBA_29_ (T^10^A^11^ and T^19^T^20^ loops) toward the bulk solution for access (binding) to thrombin. Previous reports revealed the binding of TBA_15_ and TBA_29_ to exosite I and exosite II of thrombin were through their T^3^T^4^/T^12^T^13^ loops and T^10^A^11^/T^19^T^20^ loops, respectively (Padmanabhan et al., 1993; Tasset et al., 1997).

The longest TCT time of 1,640 ± 20 s indicates that Supra-TBA_15/29_-GO prepared with GO (40 μg mL^−1^) at 60°C has the most significant impact on coagulation delay triggered by thrombin relative to the Supra-TBA_15/29_ and other Supra-TBA_15/29_-GO nanocomposites. Although Supra-TBA_15/29_-GO prepared in the lower temperature (< 60°C) possess higher TBA density on GO (Table S2), the Supra-TBA_15/29_ tend to release from GO when incubated in human plasma due to the competitive interaction between high concentrated plasma components (e.g., serum albumin) and A_20_ of Supra-TBA_15/29_ (Figure S9, Supporting Information) (Lu et al., 2016). We speculated that only a small portion of the A_20_ of the highly dense Supra-TBA_15/29_ was adsorbed on GO and thus they were easily released from GO in complicated plasma. As a result, the anticoagulation ability was lower when the Supra-TBA_15/29_ were prepared at 25 and 40°C. On the other hand, the Supra-TBA_15/29_ probably disassembled when the Supra-TBA_15/29_-GO was prepared at 90°C and resulted in much weaker anticoagulation activity than that of prepared at 60°C. The optimized Supra-TBA_15/29_-GO prepared with GO (40 μg mL^−1^) at 60°C exhibited the strongest inhibitory ability mainly due to an appropriate density, flexibility, and orientation of multivalent TBA on GO. All these factors are controllable by carefully controlling the concentration ratio of Supra-TBA_15/29_ to GO and reaction temperature, and thereby the interaction between Supra-TBA_15/29_ and GO. The appropriate orientation of the Supra-TBA_15/29_ on the slightly reduced GO surface and the desired distance between TBA_15_ and TBA_29_ resulted in a superior multivalent binding. Compared with free TBA_15_ (*K*_d_ ~ 100 nM) and TBA_29_ (*K*_d_ ~ 0.5 nM) (Padmanabhan et al., 1993; Tasset et al., 1997), Supra-TBA_15/29_-GO prepared with GO (40 μg mL^−1^) at 60°C exhibited much higher binding affinity toward thrombin (*K*_d_ = 1.9 × 10^−11^ M, Figure S10, Supporting Information). The Supra-TBA_15/29_-GO prepared with GO (40 μg mL^−1^) at 60°C also exhibited superior anticoagulation activity compared to our previously reported bivalent TBA_15_/TBA_29_-modified gold nanoparticles and GO (Huang et al., 2016b; Lai et al., 2018), probably because the particular flexible conformation and multivalency of Supra-TBA_15/29_ structure on GO boosted their anticoagulation potency. A higher concentration of GO (80 μg mL^−1^) for the preparation of Supra-TBA_15/29_-GO prepared at 60°C leads to lower aptamer density and lower local concentration on its surface, and lower concentration of GO (20 μg mL^−1^) might cause steric hindrance for thrombin binding due to undesired conformation of highly dense aptamers on its surface. On the other hand, the Supra-TBA_15/29_-GO prepared at 90°C with 20 μg mL^−1^ of GO exhibits a slightly better anticoagulant activity in comparison with that of 40 and 80 μg mL^−1^ of GO. The slightly higher anticoagulant activity could be ascribed to the higher dense aptamers on the surface of GO with a concentration of 20 μg mL^−1^ (Table S2, Supporting Information). The optimized Supra-TBA_15/29_-GO prepared with GO (40 μg mL^−1^) at 60°C was used throughout the experiment.

**Figure 3:**
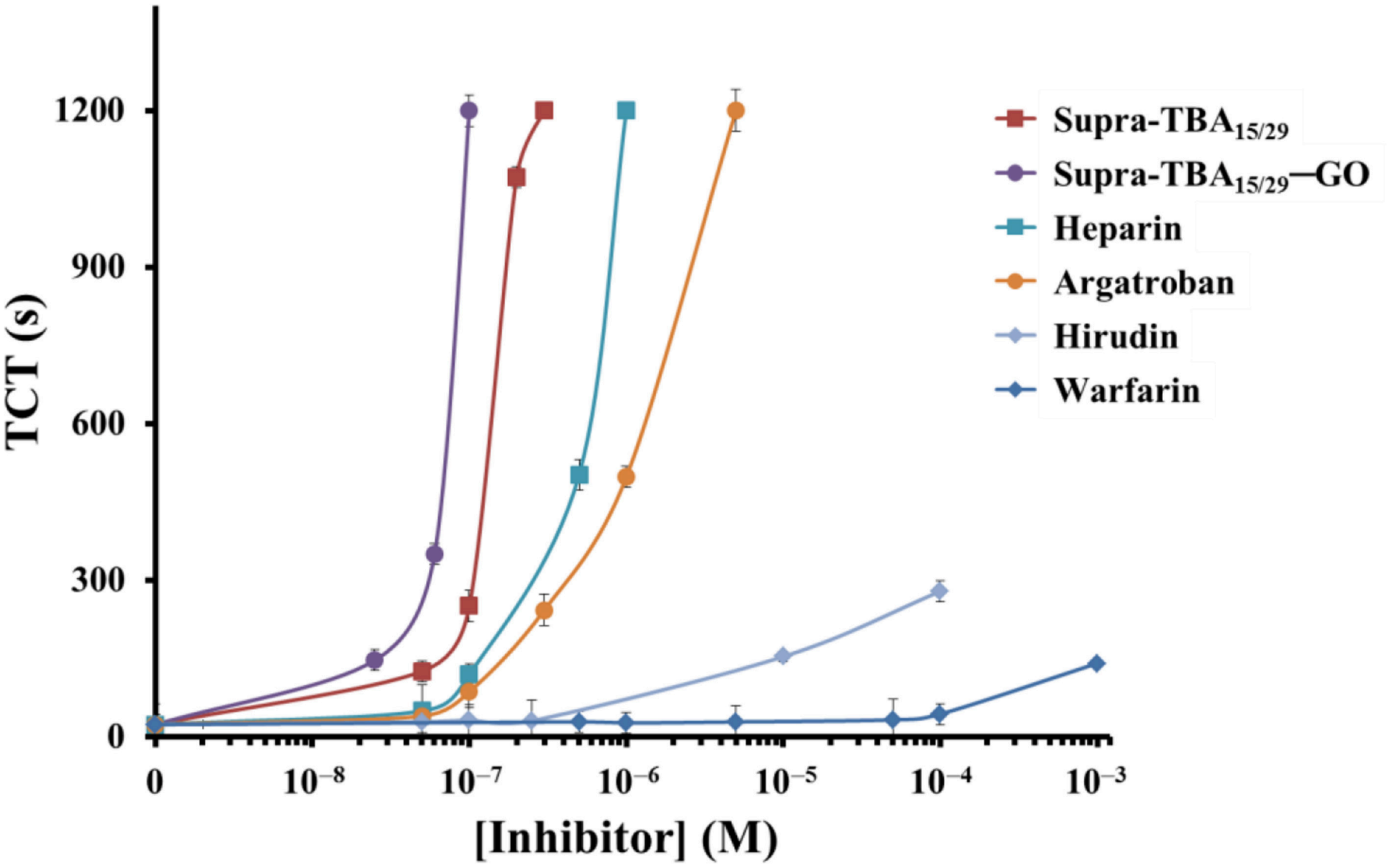
Dose-dependence of the TCTs in human plasma in the presence of Supra-TBA_15/29_, Supra-TBA_15/29_-GO and commercial drugs heparin, argatroban, hirudin, and warfarin. The longest clotting assay time recorded was set at 1,200 s. Error bars represent the standard deviations of experiments in triplicate. Other conditions were the same as those described in Figure 1.

The dose-dependent TCT displayed in Figure 3 clearly demonstrates that optimized Supra-TBA_15/29_-GO has the highest inhibition of thrombin activity, in comparison with free Supra-TBA_15/29_ and the four tested commercial anticoagulant drugs including heparin, argatroban, hirudin, and warfarin. The TCT delay caused by Supra-TBA_15/29_-GO ([TBA] = 100 nM) was 5, 10, 14, 37, and 40 times longer than that caused by the Supra-TBA_15/29_, heparin, argatroban, hirudin, and warfarin (0.1 μM), respectively. The TCT assays clearly indicated that the clotting time delay by Supra-TBA_15/29_-GO was much longer than that of Supra-TBA_15/29_ and the commercial anticoagulants. Our results reveal that Supra-TBA_15/29_-GO is a stable and highly inhibitive nanocomposite for thrombin through elaborative construction of supramolecular TBA structures on the GO surface.

### Thromboelastography

**Figure 4:**
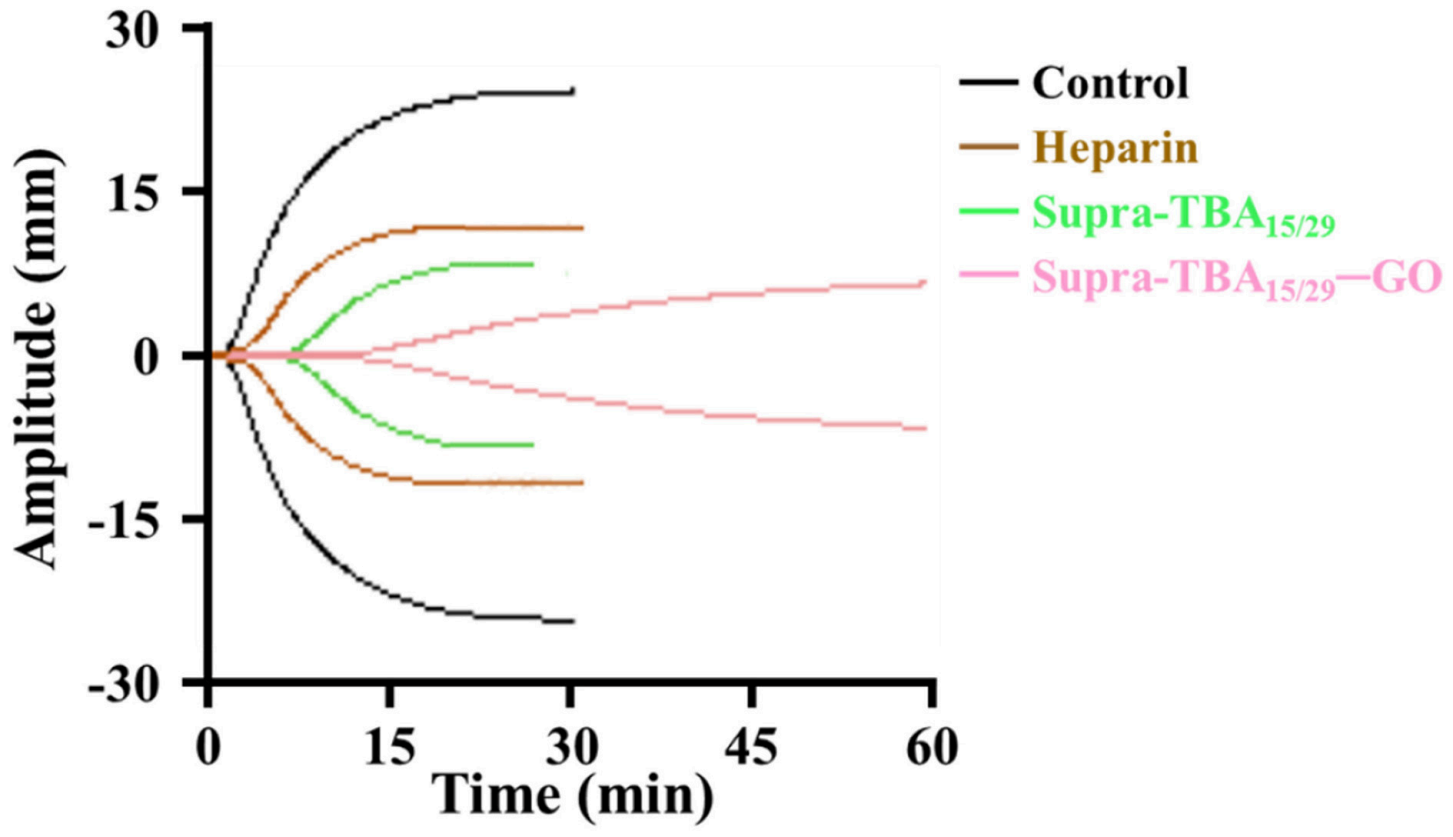
Thromboelastography (TEG) of human whole blood treated with PBS control, heparin (100 nM), Supra-TBA15/29 (100 nM), and Supra-TBA15/29-GO (100 nM in term of TBA). N/A, not available.

Platelets are tiny blood cell fragments that play an important role in blood clotting, including being activated to provide assembly sites for coagulation factor complex formation, combining with the fibrin clot and releasing agonists to amplify the platelet responses (De Candia, 2012). The evaluation of inhibition efficiency of Supra-TBA_15/29_-GO by TCT assay has limitations, because they are conducted in plasma without platelets (thrombocytes). Therefore, we employed thrombin-activated thromboelastography (TEG) to study the kinetics of inhibition of clot formation in whole blood. Various parameters, such as the R time (time of latency from start of test to initial fibrin formation, amplitude of 2 mm), K time (time taken to achieve a certain level of clot strength, amplitude of 20 mm), α angle (measuring the speed at which fibrin build up and cross linking takes place), and MA (the ultimate strength of the fibrin clot) were measured using the TEG assay (Bolliger et al., 2012) to quantify the blood clot formation. As shown in Figure 4, Supra-TBA_15/29_ ([TBA] = 100 nM) prolonged the R time (4.7 ± 0.4 min) as compared with the control (without inhibitor) (1.0 ± 0.1 min), whereas Supra-TBA_15/29_-GO prolonged the R value to 9.8 ± 0.6 min revealing that Supra-TBA_15/29_ exhibited a better inhibitory effect after it conjugated with GO. This result also indicates that Supra-TBA_15/29_-GO in the whole blood sample can inhibit the coagulation pathway chain reaction more effectively than heparin (the most widely used anticoagulant) which prolonged the R value to 2.0 ± 0.2 min. Our results reveal that the anticoagulant activity of Supra-TBA_15/29_-GO is about 5 times better than heparin in native human blood on the basis of R time values. Further, compared to the α angle, K time, and MA of Supra-TBA_15/29_ and heparin, Supra-TBA_15/29_-GO has the longest K time and smallest α angle and MA values, indicating that the clot (fibrinogen polymerization and platelet aggregation) formed has the lowest rate and strength.

### Biocompatibility

Carbon nanomaterials are reported to possess good biocompatibility (Seabra et al., 2014; Bhattacharya et al., 2016; Ou et al., 2016). Although GO has been shown to cause the rupture of cell membrane, the magnitude of decrease in cell viability does not exceed 20% for 24 h or even longer time when the concentration of GO is < 100 μg mL^−1^ (Liao et al., 2011; Fiorillo et al., 2015). Additionally, the cytotoxicity of GO is highly diminished after capping with biopolymers, such as proteins, oligonucleotide, and polysaccharide (Chong et al., 2015; Rezaei et al., 2016; Kenry, 2018; Liu et al., 2018; Ren et al., 2018). In this study, we used MTT assay to evaluate the cytotoxicity of Supra-TBA_15/29_-GO toward different mammalian cells (Figure S11A, Supporting Information). Supra-TBA_15/29_-GO did not show any cytotoxic effect toward human lung adenocarcinoma epithelial cell (A549), human liver cancer cell (Hep-G2), human umbilical cord vein endothelial cell (HUVEC), and human embryonic kidney cell (HEK293T). The cells showed cell viability of >95% even at 1.00 μM (in terms of TBA) TBA_15/29_-GO concentration and 24 h of incubation. It is noteworthy to mention that the concentration of Supra-TBA_15/29_-GO used for MTT assay was several times higher than the effective concentration of Supra-TBA_15/29_-GO used for antithrombin activity in plasma (Figures 2, 3) and whole blood (Figure 4). Live/dead cell viability staining (Calcein AM/EthD-1) was further employed for examining live and dead cells. Different concentrations of Supra-TBA_15/29_-GO ([TBA] = 0.01–1.00 μM) were added into a 24-well plate containing HEK293T cells. The morphologies of the HEK293T cells after 24 h culture with different Supra-TBA_15/29_-GO concentrations showed no obvious differences when compared to the control (Figure S11B, Supporting Information). Supra-TBA_15/29_-GO-treated cells were similar to that of the control group (PBS-treated only) which displayed typical fibroblast-like morphology (Yan and Shao, 2006). Calcein AM is a non-fluorescent dye that can easily permeate into live mammalian cells with an intact cell membrane. The hydrolysis of calcein AM by intracellular esterases produces calcein, which can be well-retained in the cell cytoplasm. Calcein exhibits strong green fluorescence at 520 nm upon excitation at 480–500 nm. EthD-1 cannot permeate through intact plasma membranes of live cells, but can enter cells with damaged cell membranes, and exhibits a strong red fluorescence (~40-fold) at ~635 nm at excitation wavelengths of 480–500 nm when it binds to nucleic acids in dead cells. The live/dead cell viability after calcein AM/EthD-1 staining further proved the low cytotoxicity of Supra-TBA_15/29_-GO, with concentrations as high as 1.00 μM and with green fluorescent cells predominating in the population (>98%). In addition, *in vitro* hemolysis experiments with defibrinated red blood cells (RBCs) did not show significant hemolysis for varying concentrations of Supra-TBA_15/29_-GO (0–1.00 μM; Figure S12, Supporting Information). Overall, our results reveal that Supra-TBA_15/29_-GO has good biocompatibility and low cytotoxicity toward mammalian cells.

### *In vivo* Rat-Tail Bleeding Assay

**Figure 5:**
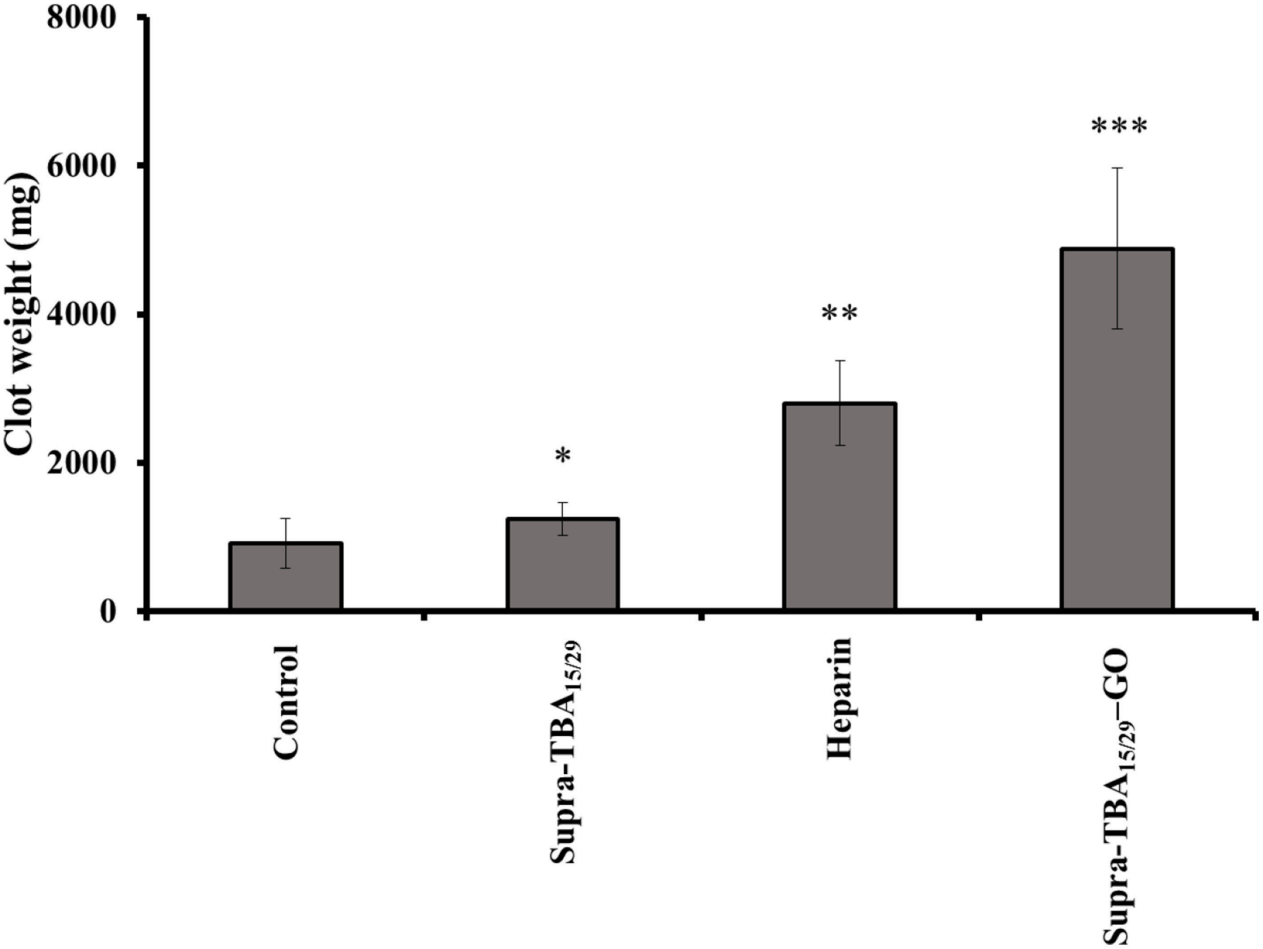
The effect of Supra-TBA_15/29_, heparin, and Supra-TBA_15/29_-GO on rat-tail bleeding. Blood clots were collected after intravenous administration of the inhibitors (2.0 μM, 100 μL). Error bars represent the standard deviations of experiments in five rats. An asterisk indicates statistically significant differences (^*^P < 0.05, ^**^P < 0.01, ^***^P < 0.001; *n* = 5) as compared with the control group.

Tail-bleeding assay in rat was performed to understand the anti-hemostatic effect of Supra-TBA_15/29_-GO *in vivo*. The rats (~200–250 g) were dosed (50 μL/100 g) by intravenous injection with heparin (2.0 μM), Supra-TBA_15/29_ (2.0 μM), or Supra-TBA_15/29_-GO (2.0 μM) and waited for 5 min. Then, the rat tails were fully transected 4 mm from the tip. The control group of rats dosed with PBS had an average blood clot weight of 918.6 ± 1.2 mg (*n* = 5), as shown in Figure 5. Compared with heparin, the Supra-TBA_15/29_-GO-treated group showed superior anticoagulant effect. The blood clot weights of the Supra-TBA_15/29_-GO-treated group (4,879 ± 900 mg; *n* = 5) were significantly heavier than that of the control group (*P* < 0.001) and the heparin-treated group (*P* < 0.05). The body weights of the Supra-TBA_15/29_-GO treated rats were almost the same as those of the untreated group (*P* > 0.05, *n* = 5) 10 days post-dose (data not shown). In addition, all Supra-TBA_15/29_-GO treated rats survived for the next 2 months and exhibited normal behavior. The *in vivo* rat-tail bleeding assay study indicated that highly biocompatible Supra-TBA_15/29_-GO possesses great potential as a safe and efficient anticoagulant nanodrug for the treatment of thrombotic diseases, such as deep venous thrombosis, myocardial infarction, and thrombotic stroke.

## Conclusions

In this study, highly stable and biocompatible supramolecular-aptamer functionalized GO nanosheets were prepared by a biomimetic approach. We successfully designed two different aptamers that can connectively hybridize and self-assemble on GO. AFM images showed that the Supra-TBA_15/29_-GO was dispersed as 2D nanosheets. The Supra-TBA_15/29_-GO was stable in high ionic strength solution as well as in human plasma. The structure of Supra-TBA_15/29_ on GO is controllable by mediating the ratio of Supra-TBA_15/29_ to GO and preparation temperature. The efficient inhibitory activity of Supra-TBA_15/29_-GO against thrombin is due to precisely programmed TBA_15_ and TBA_29_'s hybridized structure on GO leading to strong interactions with thrombin and steric hindrance from fibrinogen substrate. The dose-dependent TCT delay caused by Supra-TBA_15/29_-GO was >10 times longer than that of the most widely used anticoagulant heparin. In addition, the TEG and rat-tail bleeding experiment further proved the superior anticoagulant activity of Supra-TBA_15/29_-GO relative to heparin. In the future, GO with small sizes may be employed for preparing Supra-TBA_15/29_-GO to more efficiently reduce the uptake from the reticuloendothelial system in animals. In addition, various aptamers which target with different coagulation factors can be co-programmed on GO for systematic inhibition of multiple coagulation factors. Moreover, bioaccumulation, biopsy, metabolism, and acute and chronic toxicity should be conducted in animal models to confirm the potential of supramolecular aptamer-GO nanocomposites as a safe and viable drug.

## Experimental Methods

### Synthesis and Characterization of GO

GO was prepared by a modified Hummer's method (Hummers and Offeman, 1958; Marcano et al., 2010). Briefly, concentrated sulfuric acid (90 mL) and concentrated phosphoric acid (10 mL) were mixed and added to graphite powder (0.75 g) in a round bottomed flask. Potassium permanganate (4.50 g) was slowly added to the mixture and maintained at 50°C for 12 h with continuous stirring. Then, the reaction mixture was allowed to cool and placed in an ice bath, followed by slowly adding deionized water (100 mL) and hydrogen peroxide (32%) to the mixture until the solution turned from dark purple to bright yellow. The supernatant was removed by centrifugation at 35,000 *g* for 30 min. The residue was washed repeatedly with 5 mM sodium phosphate buffer (pH 7.4) until the solution pH was close to 7. The as-obtained graphite oxide was exfoliated by sonication for 2 h (power 200 W). The unoxidized graphite and large particles were separated at a relative centrifugal force (RCF) of 15,000 *g* for 30 min, and its weight/volume concentration was calculated by lyophilisation. An atomic force microscope (Shimadzu SPM-9600 AFM, Shimadzu Co, Kyoto, Japan) was used to analyze the size of GO. The dynamic light scattering (DLS) and zeta potential experiments were performed using a Zetasizer 3000HS analyzer (Malvern Instruments, Malvern, UK). X-ray photoelectron spectroscopy (XPS) was performed using an ES-CALAB 250 spectrometer (VG Scientific, East Grinstead, UK) with Al Kα X-ray radiation as the X-ray excitation source. Binding energies were corrected using the C1s peak at 284.8 eV as the standard. We also analyzed GO at different degrees of oxidation by using a DXR Raman microscope (Thermo Fisher Scientific Inc., Waltham, MA, USA) equipped with a 50X objective, a Nd:YAG laser (532 nm) and a charge-coupled detector. FT-IR spectroscopy was performed by using a Cary 640 FT-IR spectrometer (Santa Clara, CA, USA).

### Synthesis of Supra-TBA_15/29_-GO

A_20_h_15_T_5_TBA_15_T_5_h_15_ (sTBA_15_) and h_15_T_5_TBA_29_T_5_h_15_A_20_ (sTBA_29_) (2.5 μM) were allowed to hybridize at 4°C for 1 h in PBS to form stable Supra-TBA_15/29_. GO (40 μg mL^−1^) was added to the Supra-TBA_15/29_ and allowed to react. After 2 h of reaction, NaCl solution (1 M) was added and maintained at 4°C for 1 h. Finally, the solution was heated to 25, 45, 60, and 90°C and kept for 1 h. The resulting solution was centrifuged at an RCF of 35,000 *g* for 2 h and the residues were resuspended with PBS. After three centrifugation and washing cycles, the amount of Supra-TBA_15/29_ adsorbed on GO was determined by quantitation of the un-adsorbed TBA in the supernatants by using Quant-iT™ OliGreen ssDNA Reagent. The material was stored at 4°C when not in use and found to be stable for up to 3 months.

### Thrombin Clotting Time (TCT) Assay

TCT tests for the common coagulation pathway were performed with nh-TBA_15/29_, di-TBA_15/29_, Supra-TBA_15/29_, Supra-TBA_15/29_-GO, and four commercial anticoagulants (heparin, argatroban, hirudin, and warfarin). Analytical solution containing PBS (pH 7.4), bovine serum albumin (100 μM), inhibitor (100 nM), and human plasma (2-fold diluted) was allowed to react for 15 min, maintained at 37°C for 3 min, and then mixed with thrombin (15 nM). Scattered light intensity at 650 nm was recorded using an FP-6500 spectrophotometer (JASCO, Tokyo, Japan). The TCT was noted as the time at which the differential scattering signal intensity reached the maximum. The measurements were done in triplicates, and a single batch of plasma was used for each set of experiments.

### Thromboelastography

The anticoagulation efficiency of Supra-TBA_15/29_, Supra-TBA_15/29_-GO and heparin in whole blood clots was evaluated by thromboelastography (Haemoscope corporation, Niles, IL, USA), which measures the progress of blood clot formation and platelet-fibrin bond strength and monitors the internal interactions in blood and the contributions of cellular content. The plasma samples from healthy volunteers with were drawn from the vein, transferred to tubes containing sodium citrate, and immediately centrifuged at a relative centrifugal force (RCF) of 3000 g (10 min, 4°C). The human plasma collection procedure was approved by the Chang Gung Memorial Hospital institutional review board (IRB-103-6474A3) and informed consent was obtained from the volunteers prior to the collection of the plasma. Prior to the experiment, plain disposable plastic TEG cups (Haemonetics) were maintained at 37°C. In total, 52 μL of anticoagulant (0.5 μM) in PBS and 288 μL of whole blood were mixed in a TEG cup and incubated at 37°C for 10 min. To initiate the whole blood coagulation at 37°C, 20 μL of thrombin solution (270 nM) was added to the above mixture. The clot formation was recorded until a stable clot was formed or 1 h had passed. The various parameters, such as the reaction kinetics, α angle, and maximum amplitude were calculated using the TEG® Analytical Software version (TAS) 4.2.3 (Haemonetics).

### Rat-Tail Bleeding Time

We used rat tail bleeding time to compare the anticoagulant effect of inhibitors *in vivo* in male rats of the Sprague Dawley (SD) strain weighing between 200 and 250 g. The experiments were conducted after getting permission from the Institutional Animal Care and Use Committee of the National Laboratory Animal Center (Permit number License No. IACUC106049). The rats were anesthetized with Zoletil 50 with a dose of 100 μL/100 g body weight via subcutaneous injection. Supra-TBA_15/29_ (2 μM), Supra-TBA_15/29_-GO (2 μM; in terms of TBA) or heparin (2 μM) was administered by intravenous injection at a dose of 50 μL/100 g, followed by waiting for 5 min. Then, 4 mm of the rat tail-tip was cut off, and the tail was immersed in PBS at 37°C. The blood clot weight until cessation of bleeding was determined.

### Statistical Analysis

Student's *t*-test was performed and *P*-values < 0.05 were considered significant. The probability of rat survival was determined by the Kaplan–Meier method.

See the Supporting Information for the details on the materials, determination of the binding constant of thrombin with Supra-TBA_15/29_-GO, *in vitro* cytotoxicity assays, and hemolysis assays.

## Ethics Statement

The animal experiments were conducted after getting permission from the Institutional Animal Care and Use Committee of the National Laboratory Animal Center (Permit number License No. IACUC106049).

## Author Contributions

C-CH conceived the original idea, supervised the project from start to finish, and supervised the manuscript preparation. T-XL and P-XL carried out the experiments. J-YM and H-WC provided feedback, interpretation of the results, and helped with the preparation of the manuscript. BU and AA helped with manuscript preparation. All authors discussed the results and contributed to the manuscript.

### Conflict of Interest Statement

The authors declare that the research was conducted in the absence of any commercial or financial relationships that could be construed as a potential conflict of interest.
