# Supplementary material for: Supramolecular Aptamers on Graphene Oxide for Efficient Inhibition of Thrombin Activity
Source: Front Chem. 2019 May 16;7:280. doi: 10.3389/fchem.2019.00280 (PMC6532589; doi:10.3389/fchem.2019.00280)
Supplement: Supplementary file 1 [file Data_Sheet_1.docx]

***Supporting Information***

**Supramolecular Aptamers on Graphene Oxide for Efficient Inhibition of Thrombin Activity**

*Ting-Xuan Lin,^1^ Pei-Xin Lai,^1^ Ju-Yi Mao,^1,2,3^ Han-Wei Chu,^1^ Binesh Unnikrishnan,**^1^ Anisha Anand ^1^ and Chih-Ching Huang^1,4,5*^*

*^1^ Department of Bioscience and Biotechnology, National Taiwan Ocean University, Keelung 20224, Taiwan*

*^2^ Doctoral Degree Program in Marine Biotechnology, National Taiwan Ocean University, Keelung 20224, Taiwan*

*^3^ Doctoral Degree Program in Marine Biotechnology, Academia Sinica, Taipei 11529, Taiwan*

*^4^ Center of Excellence for the Oceans, National Taiwan Ocean University, Keelung 20224, Taiwan*

*^5^ School of Pharmacy, College of Pharmacy, Kaohsiung Medical University, Kaohsiung 80708, Taiwan*

**Correspondence:** Professor Chih-Ching Huang, Department of Bioscience and Biotechnology, National Taiwan Ocean University, 2 Beining Road, Keelung 20224, Taiwan; Tel.: 011-886-2-2462-2192 ext. 5517; E-mail: huanging@ntou.edu.tw

**Supplementary Experimental Section:**

**Materials.** Graphite powder (99%, 7−11 μm) and hydrogen tetrachloroaurate(III) trihydrate (HAuCl_4_•3H_2_O) were purchased from Alfa Aesar (Heysham, Lancashire, UK). Potassium permanganate, sulfuric acid, 32% hydrogen peroxide were purchased from SHOWA (Tokyo, Japan). Phosphoric acid and acetic acid were purchased from Mallinckrodt Baker (Phillipsburg, NJ, USA). Sodium phosphate, calcium chloride (CaCl_2_), magnesium chloride (MgCl_2_), potassium chloride (KCl), sodium chloride (NaCl), trisodium citrate, human α-thrombin (1,000 NIH units/mg protein), human fibrinogen, bovine serum albumin (BSA), argatroban, and hirudin were purchased from Sigma-Aldrich (Milwaukee, WI, USA). Warfarin was purchased from Fluka (USA). Heparin was purchased from Merck (Darmstadt, Germany). Trisodium citrate dihydrate was purchased from J. T. Baker (USA). OliGreen was purchased from Thermo Fisher Scientific. Ultrapure water from a Mili-Q Simplicity^TM^ 185 system with the resistivity no less than 18.2 MΩ·cm was used for all experiments. Phosphate-buffered saline (PBS; containing 25.0 mM tris-HCl, 150.0 mM NaCl, 5 mM KCl, 1.0 mM MgCl_2_ and 1.0 M CaCl_2_; adjusted to pH 7.4 using HCl) was used to mimic physiological conditions.

**Binding Constant.** The dissociation constant (*K*_d_) of Supra-TBA_15/29_−GO and thrombin was determined by adding aliquots of each Supra-TBA_15/29_−GO solution ([Supra-TBA_15/29_] = 100 pM) with different concentrations of thrombin (0−1 nM) in PBS, in the presence of 100 µM BSA, incubated for 30 min. After the thrombin−Supra-TBA_15/29_−GO complex was formed, the solution was centrifuged at 35,000 *g* for 1.5 h. The supernatant containing unbound thrombin in the supernatant was estimated using fibrinogen–modified Au NPs, as reported earlier (Chen et al., 2010). To estimate the binding affinity between Supra-TBA_15/29_−GO and thrombin, Scatchard equation was used:

*N*_Thrombin_/[Free-Thrombin] = *N*_max_/*K*_d_ − *N*_Thrombin_/*K*_d_ (1)

*N*_Thrombin_ refers to the number of thrombin molecules binding to Supra-TBA_15/29_−GO at equilibrium, *N*_max_ is the number of binding sites per Supra-TBA_15/29_−GO, [Free-Thrombin] is the unbound thrombin concentration at equilibrium, and *K*_d_ is the dissociation constant. The *K*_d_ and *N*_max_ values can be extrapolated using the calculated slope and intercept of the *N*_Thrombin_/[Free-Thrombin] plot against *N*_Thrombin_.

**Cytotoxicity.** Cell viability was measured by MTT assay and LIVE/DEAD cell assay. Human lung adenocarcinoma epithelial cell (A549 cell line), human liver cancer cell (Hep-G2 cell line), human umbilical cord vein endothelial cell (HUVEC cell line), and human embryonic kidney cell (HEK293T cell line) were purchased from American Type Culture Collection Center (ATCC, Manassas, VA, USA). HUVECs are normal cell among the four cell lines. For MTT assay, the HEK293T, Hep-G2, and A549 cells were maintained in a cell culture chamber dissolved in DMEM medium. The medium was supplemented with 10% Fetal Bovine Serum, 1% ampicillin (antifungal antibiotics), 2.0 mM L-Glutamine, and 1% Non-essential amino acids in 5% CO_2_ at 37 ºC. HUVECs were routinely cultured in tissue culture flasks in vasucLife EnGS ECG medium at 37 °C humidified atmosphere containing 95% air and 5% CO_2_. A549, Hep-G2, HUVEC, and HEK293T cell lines were cultured in 48-well plates for 24 h (*ca.* 5.0 × 10^4^ cells/well). Subsequently, the culture solution was replaced with a culture solution containing Supra-TBA_15/29_−GO (0−1.0 μM; in terms of TBA) and cultured for 24 h. The supernatant was removed and added to the MTT solution (1%) and the culture medium. After 1 h of the reaction, the MTT solution and the culture solution were removed. The final product was dissolved in isopropanol (200 μL) to detect absorption at wavelength of 595 nm by Multi-Mode Microplate Reader.

LIVE/DEAD Viability/Cytotoxicity Kit (Invitrogen, USA) was further used for examining live and dead cells. The HEK293T cells were maintained in a cell culture chamber dissolved in DMEM medium at 37 °C humidified atmosphere containing 95% air and 5% CO_2_. HEK293T cell lines were cultured in 24-well plates for 24 h (*ca.* 3.0 × 10^5^ cells/well). Subsequently, the culture solution was replaced with a culture solution containing Supra-TBA_15/29_−GO (0−1.0 μM; in terms of TBA) and cultured for 24 h. The HEK293T cell cultures, after washing three times with PBS, were stained with a working solution consisting of PBS (1 mL), EthD-1 (2 μL), and calcein AM (0.5 μL) and viewed under a fluorescence microscope (Axiovert 200 M, Carl Zeiss, Oberkochen, Germany).

**Hemolysis Assay.** Fresh blood was collected from a healthy volunteer (25 years) using a blood collection tube containing ethylenediaminetetraacetic acid (EDTA). The collected sample was centrifuged at 3000 *g* for 10 min at 4 ºC to separate the serum from the red blood cells (RBCs). The RBCs were diluted with sterile isotonic physiological buffer to obtain an RBC stock suspension (*ca*. 4 vol.% of blood cells). The RBC stock suspension (100 μL) was added to each Supra-TBA_15/29_−GO (0−1.0 μM; in terms of TBA) in PBS in 1.5 mL vials and incubated at 37 ºC for 1 h with shaking at 160 rpm. Each of the mixture was centrifuged at an RCF of 4000 *g* for 5 min. Hemolysis activity was determined by measuring hemoglobin absorption at 576 nm (OD_576_) in the supernatant (200 μL). The sterile isotonic physiological buffer was used as a reference for 0% hemolysis (OD_576 blank_). The one hundred percent hemolysis control was made by adding ultrapure water to the RBC suspension (OD_576 ultrapure water_). The hemolysis activity was calculated as follows:

Hemolysis (%) = [(OD_576 supra-TBA15/29–GO_ − OD_576 blank_)/(OD_576_ _ultrapure water_ − OD_576 blank_)] × 100

**Reference:**

Chen, C.-K., Huang, C.-C., and Chang, H.-T., (2010) Label-free colorimetric detection of picomolar thrombin in blood plasma using a gold nanoparticle-based assay. *Biosens. Bioelectron.* 2010, 25, 1922–1927. doi:10.1016/j.bios.2010.01.005

**Table S1.** DNA sequence of thrombin-binding aptamers used in this study.

| **Name** | **Sequence*^a^*** |
| --- | --- |
| **sTBA_15_ (**A_20_h_15_T_5_TBA_15_T_5_h_15_) | 5’-AAA AAA AAA AAA AAA AAA AAC GAC CTG TAC GAC TCT TTT TTCA GGG TTG GTG TGG TTG GCT GAT TTT TATC TTC ACG AGC ACT-3’ |
| **sTBA_29_**  **(**h_15_T_5_TBA_29_T_5_h_15_A_20_) | 5’-GAG TCG TAC AGG TCG TTT TTA GTC CGT GGT AGG GCA GGT TGG GGT GAC TTT TTT AGT GCT CGT GAA GAT AAA AAA AAA AAA AAA AAA AA-3’ |
| **dTBA_29_**  (nh_15_T_5_TBA_29_T_5_h_15_A_20_) | 5’-CGA CTC GTG CAA CTC TTT TTA GTC CGT GGT AGG GCA GGT TGG GGT GAC TTT TTT AGT GCT CGT GAA GAT AAA AAA AAA AAA AAA AAA AA-3’ |
| **nTBA_29_**  (nh_15_T_5_TBA_29_T_5_nh_15_A_20_) | 5’-CGA CTC GTG CAA CTC TTT TTA GTC CGT GGT AGG GCA GGT TGG GGT GAC TTT TTT CTC TTC TTC TCC TCC AAA AAA AAA AAA AAA AAA AA-3’ |
| ***^a^*underline indicates hybrid pairs** | |

**Table S2.** Density of Supra-TBA_15/29_ on GO prepared at different temperature (25−90 ºC) and GO concentration (20−80 μg mL^−1^).

|  | **Preparation temperature** | | | |
| --- | --- | --- | --- | --- |
| **GO** | **25 ºC** | **45 ºC** | **60 ºC** | **90 ºC** |
| 20 μg mL^–1^ | 47.1 nmol mg^−1^ | 41.7 nmol mg^−1^ | 35.5 nmol mg^−1^ | 26.2 nmol mg^−1^ |
| 40 μg mL^–1^ | 32.1 nmol mg^−1^ | 27.5 nmol mg^−1^ | 23.0 nmol mg^−1^ | 22.6 nmol mg^−1^ |
| 80 μg mL^–1^ | 24.9 nmol mg^−1^ | 23.2 nmol mg^−1^ | 21.7 nmol mg^−1^ | 20.3 nmol mg^−1^ |

**Table S3.** Elemental analysis of GO treated at different temperature.

| **Elemental compositions^a)^** | | | | |
| --- | --- | --- | --- | --- |
| **Temperature** | **N (%)** | **C (%)** | **H (%)** | **O (%)** |
| 25 °C | 0.1 ± 0.0 | 41.7 ± 0.4 | 3.6 ± 0.1 | 54.5 ± 0.2 |
| 45 °C | 0.2 ± 0.1 | 42.6 ± 0.7 | 3.8 ± 0.1 | 53.4 ± 0.3 |
| 60 °C | 0.1 ± 0.0 | 43.3 ± 0.6 | 3.9 ± 0.1 | 52.7 ± 1.1 |
| 90 °C | 0.1 ± 0.0 | 43.4 ± 0.6 | 3.9 ± 0.1 | 52.7 ± 0.8 |
| ^a)^ Determined by elemental analysis (n = 3). | | | | |

The carbon content slight increased (from 41.7% to 43.4%) along with a loss of relative oxygen content (from 54.5% to 52.7%) of the GO with treated temperature increased from 25 ^o^C to 90 ^o^C indicate only a mild reduction of GO was occurred at higher temperature.

**
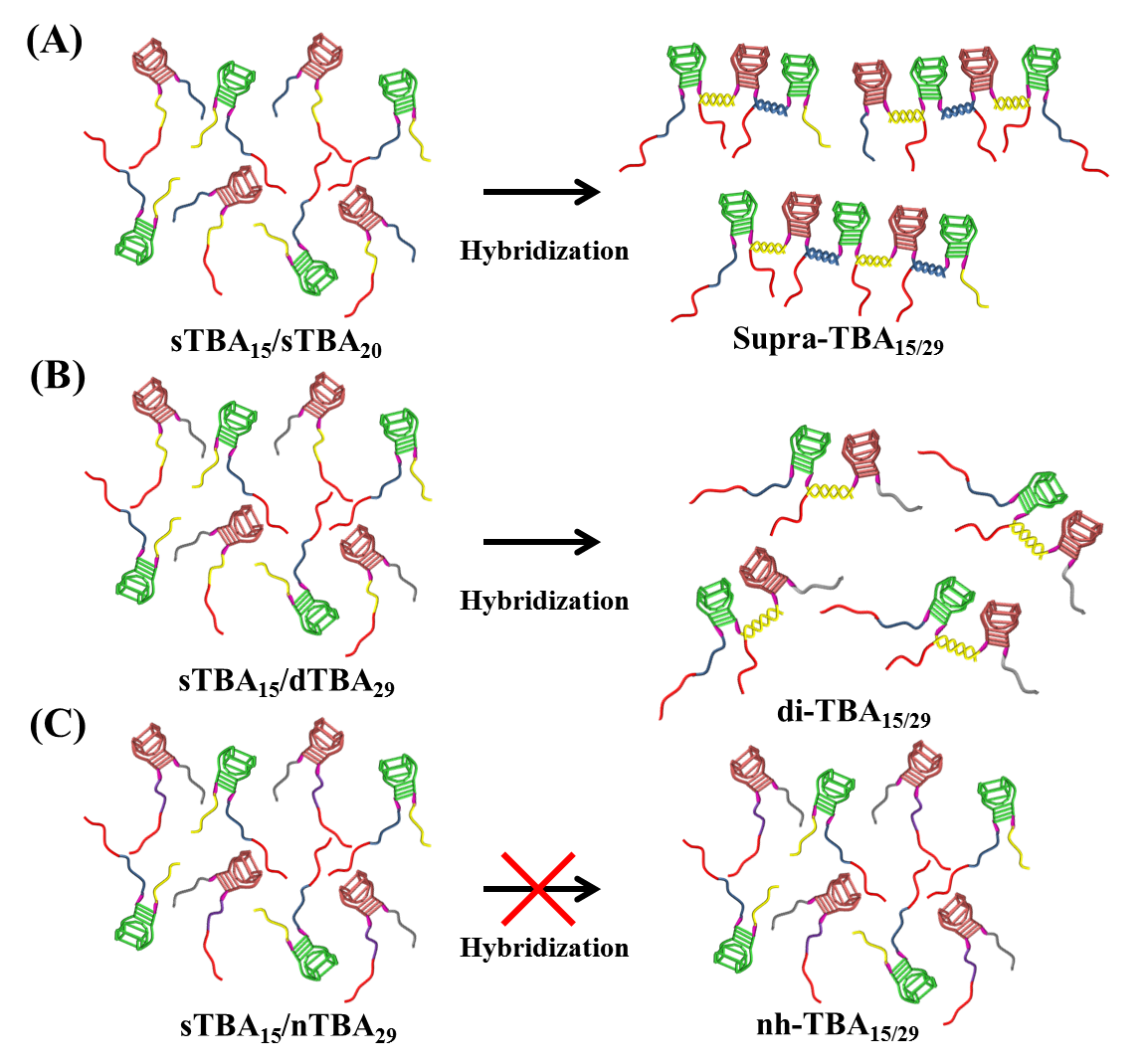
**

***Scheme S1.*** Schematic representation of the preparation of (A) Supra-TBA_15/29_, (B) di-TBA_15/29_, and (C) nh-TBA_15/29_.


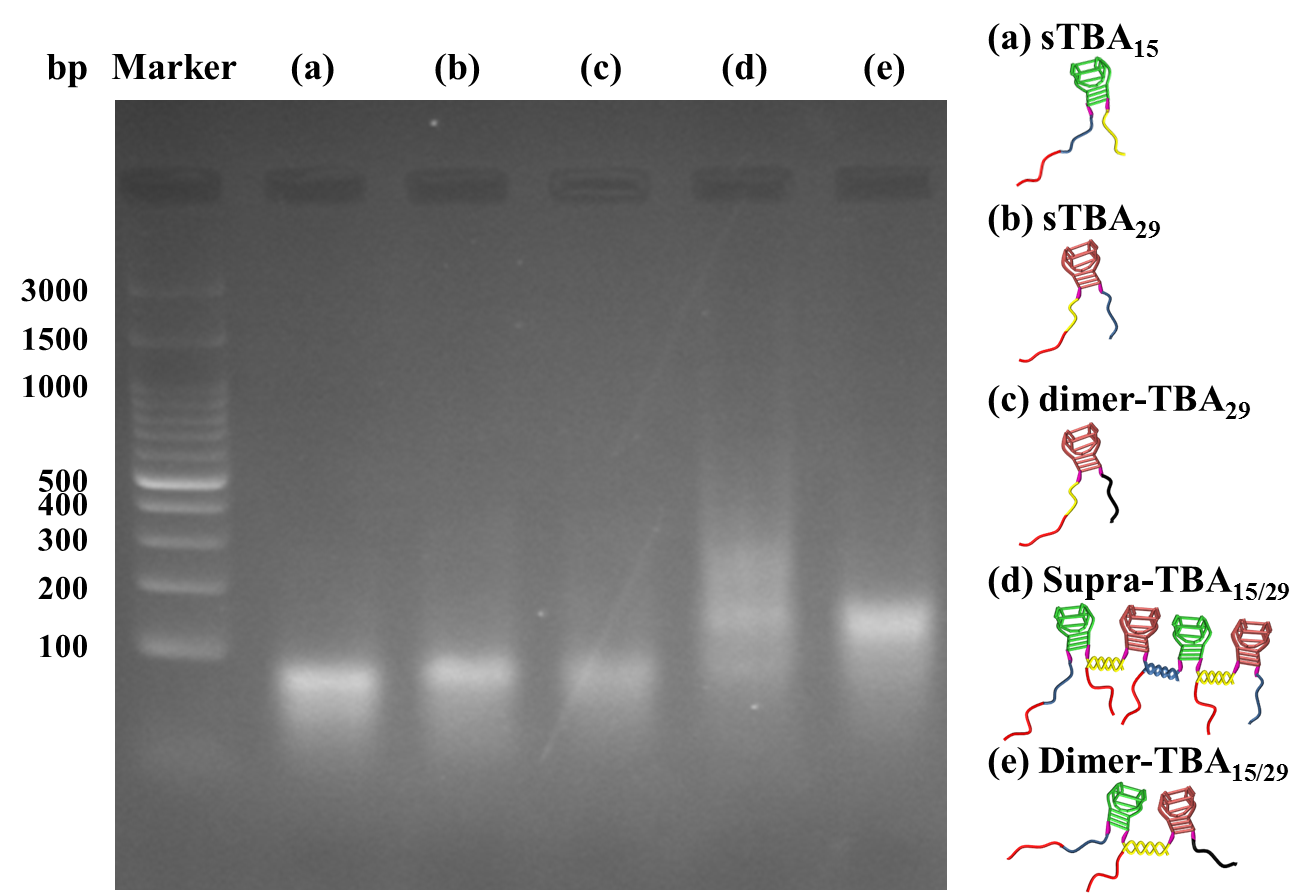


***Figure S1.*** Gel electrophoresis of (a) sTBA_15_, (b) sTBA_29_, (c) nh-TBA_29_, (d) Supra-TBA_15/29_, and (e) di-TBA_15/29_ with the concentration of 5.0 μM (in terms of TBA) in 2% agarose gel containing 100 mM tris-borate (pH 7.4) at 100 V for 30 min.

***
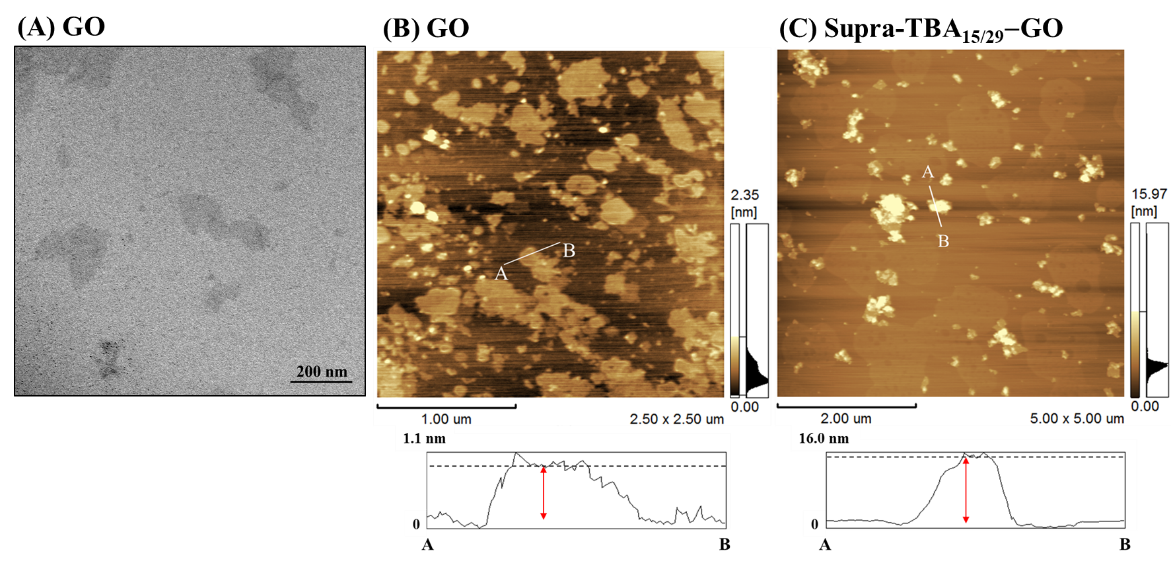
***

***Figure S2.*** (A) TEM image of GO and (B, C) tapping-mode atomic force microscope (AFM) images of (B) GO and (C) GO after modified with Supra-TBA_15/29_. The average heights of the GO and Supra-TBA_15/29_−GO as determined by AFM for (A) and (B) were ~1.1 nm and ~16.0 nm. The Supra-TBA_15/29_−GO was prepared with Supra-TBA_15/29_ ([TBA] = 2.5 μM) and GO (40 μg mL^−1^) at 60 ^o^C.

***
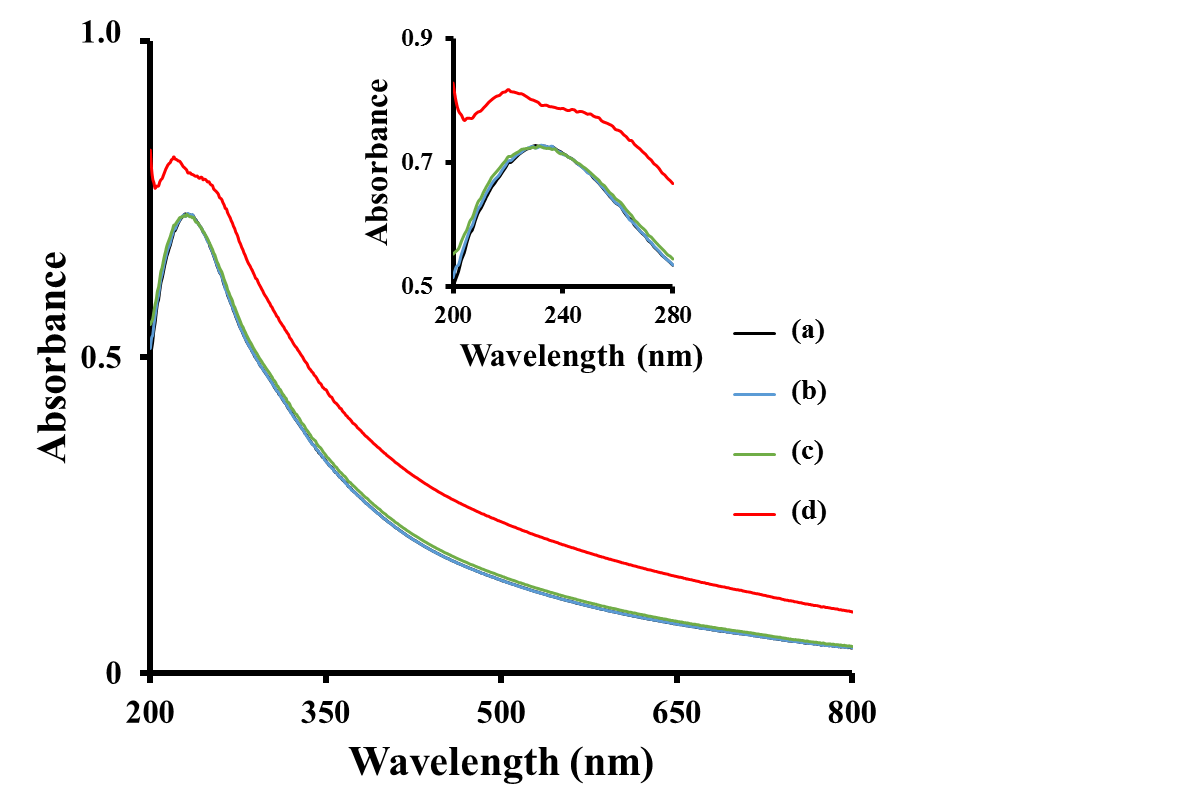
***

***Figure S3.*** UV-vis absorption spectra of GO (20 μg mL^−1^) after treated at (a) 25, (b) 45, (c) 60 and (d) 90 ºC for 2 h. The inset shows the enlarged view of the spectra in the lower wavelength region.

The absorption band of GO at 230 nm could be ascribed to π→π* electron transition in aromatic C=C in the GO plane. A smaller band at 310 nm is attributed to n→π* transition of nonbonding electrons in oxygen atoms connected with C=C bonds. The increased absorbance and shift toward higher wavelength of the band at 230 nm was observed for different GO samples, especially for that heated at 90 ºC (curve d), indicating partial reduction of GO.


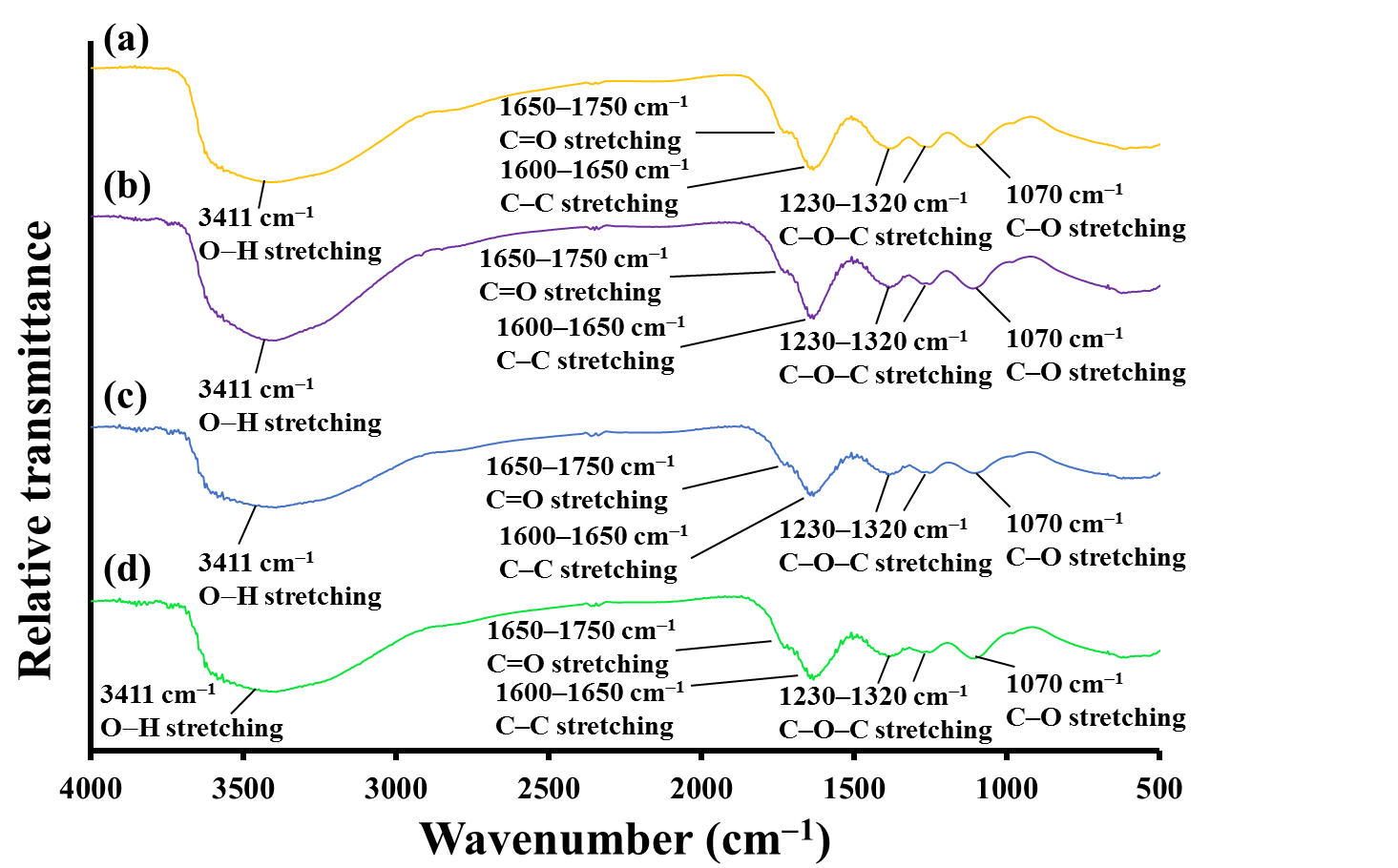


***Figure S4.*** Fourier-transform infrared (FT-IR) spectra of GO after treated at (a) 25, (b) 45, (c) 60 and (d) 90 ºC for 2 h.

The broadening of peaks for the oxygen containing functional groups of GO with the increase in temperature shows that reduction of GO has occurred with the elimination of hydroxyl, epoxy and carbonyl groups. GO exhibited a weaker C−O−C stretching peak at 1320 cm^−1^ and C−O stretching peak at 1707 cm^−1^ with the increase in temperature further suggested the higher degree of reduction of GO at higher temperature.

***
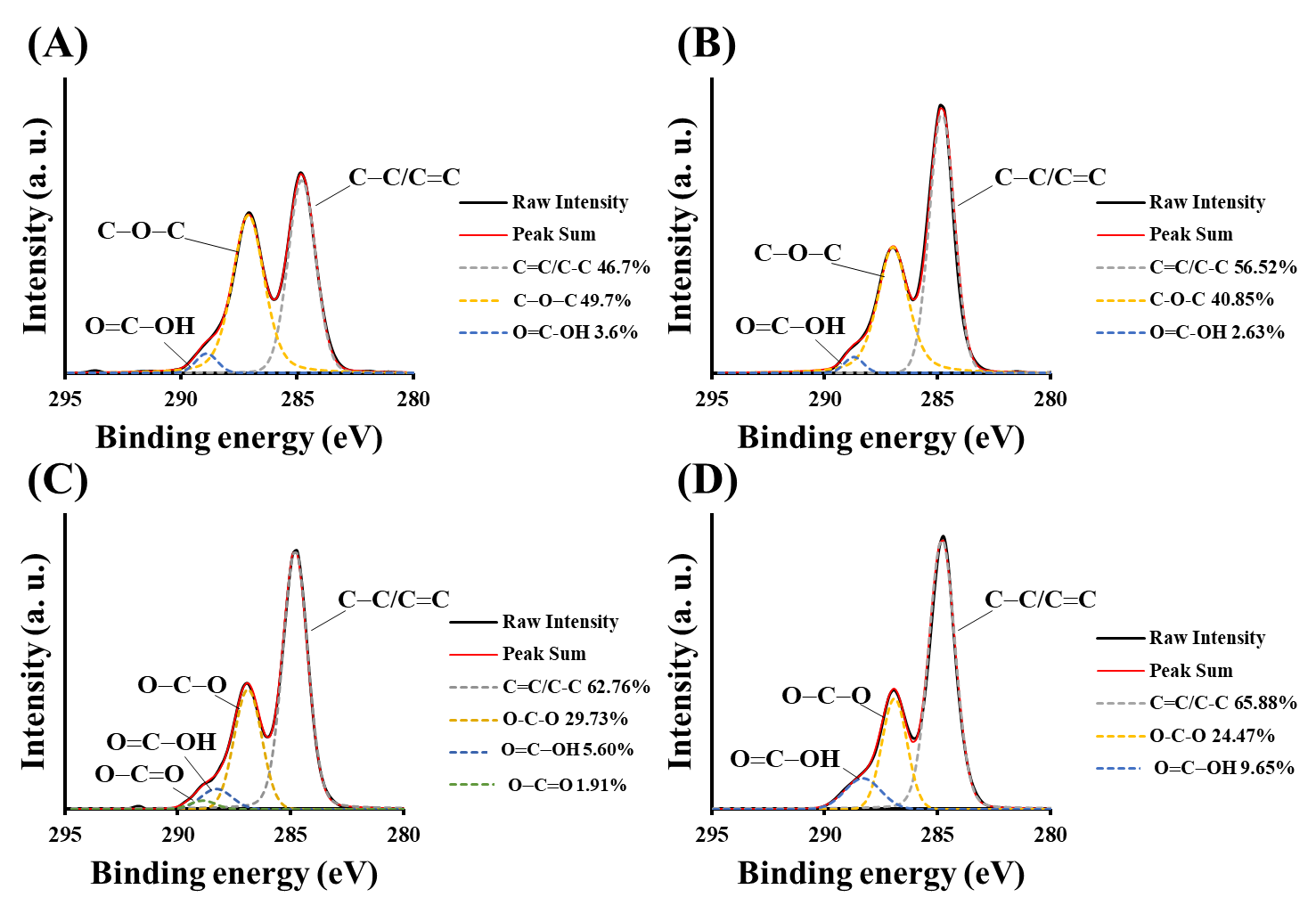
***

***Figure S5.*** X-ray photoelectron spectroscopy (XPS) C1s spectra of GO after treated at (A) 25, (B) 45, (C) 60 and (D) 90 ºC for 2 h.

XPS analysis is well-suited for the characterization of carbon-based nanomaterials and films, and is a complementary technique to Raman, FT-IR, UV-vis absorption spectroscopies and elemental analysis. Deconvolution of the C1s XPS spectra of GO revealed several carbon bonds, *viz.* C=C/C−C (284.8 eV), O−C−O (286.9 eV), C=O (287.1 eV), O−C=O (288.3 eV), and O=C−OH (288.9 eV). GO heated at 90 ºC showed greater C=C/C−C (284.8 eV, 65.88%) peak. Consistent with FT-IR, UV-vis absorption spectroscopies and elemental analysis, silight increase in C=C/C−C along with the decrease in the O−C−O reveal only a partial reduction of GO at higher temperature.

***
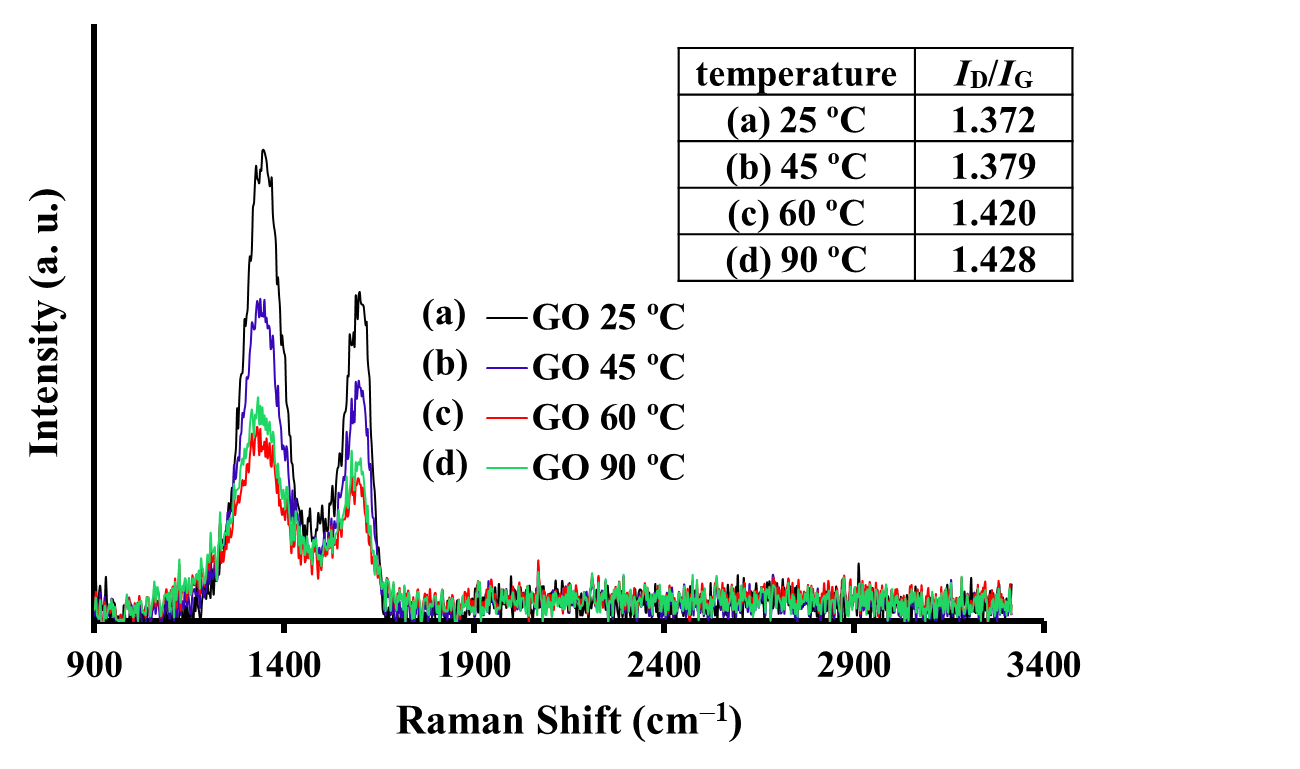
***

***Figure S6.*** Raman spectra of GO treated at (a) 25, (b) 45, (c) 60 and (d) 90 ºC for 2 h.

The intensity ratio of D and G band (*I*_D_/*I*_G_) is used to investigate the degree of disorders in graphene. This ratio also can be used to investigate surface disorder and defects of GO. After reduction of GO, two different types of region (high defect density region or low defect density region) may arise. In low defect density region, the ratio increases with the decrease of defect density. The intensity ratio (*I*_D_/*I*_G_) of GO heated at 25, 45, 60 and 90 ºC were determined to be 1.372, 1.379, 1.420 and 1.428, respectively, which further confirmed that GO was slightly reduced with increase in temperature.

**
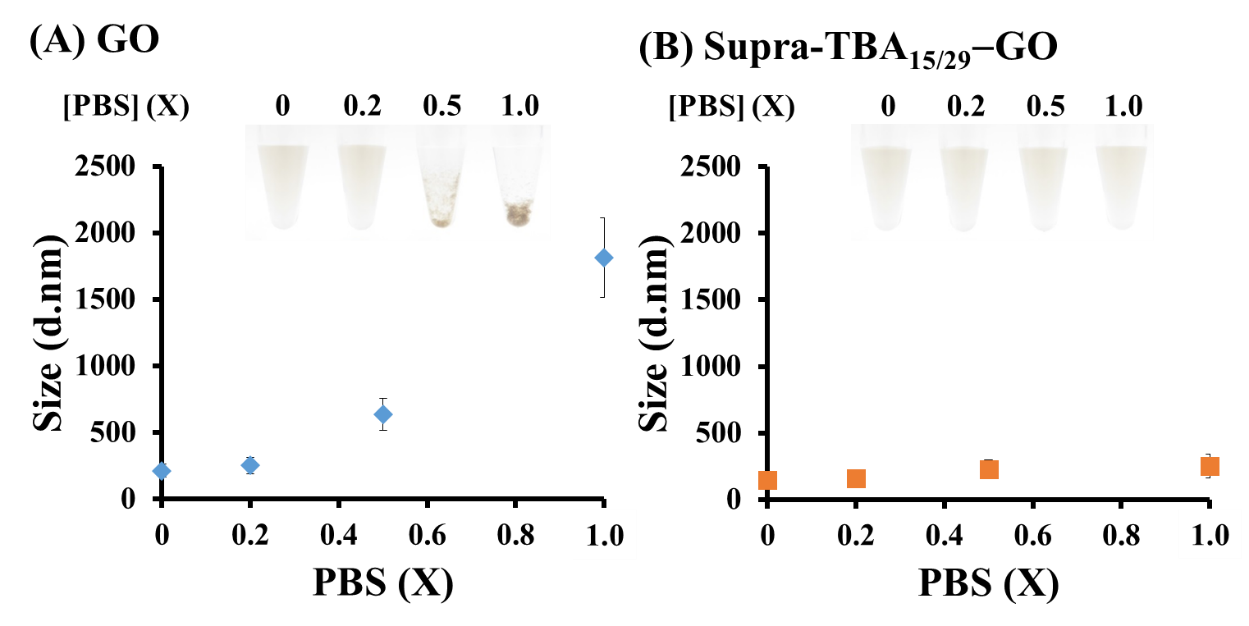
**

***Figure S7.*** Hydrodynamic size of of (A) GO (40 μg mL^−1^) and (B) Supra-TBA_15/29_−GO (40 μg mL^−1^ in terms of GO) prepared in different concentration of phosphate-buffered saline (PBS). The Supra-TBA_15/29_−GO was prepared with Supra-TBA_15/29_ ([TBA]=2.5 μM) and GO (40 μg mL^−1^) at 60 ^o^C. The inset is the photographs of the corresponding solutions. For simplicity, the concentration of PBS (containing 137 mM NaCl, 2.7 mM KCl, 10 mM Na_2_HPO_4_, and 1.8 mM KH_2_PO_4_; adjusted to pH 7.4 using HCl) is denoted as 1X. The mixture of 400 mL PBS (1X) and 600 mL H_2_O is denoted as 0.4X PBS.

All DLS spectra of other Supra-TBA_15/29_−GO prepared with different GO concentration and at different temperature exhibited very similar results to that shown in **Figure S7B**, indicating all Supra-TBA_15/29_−GO are stable (no aggregation) when incubated in PBS solution.


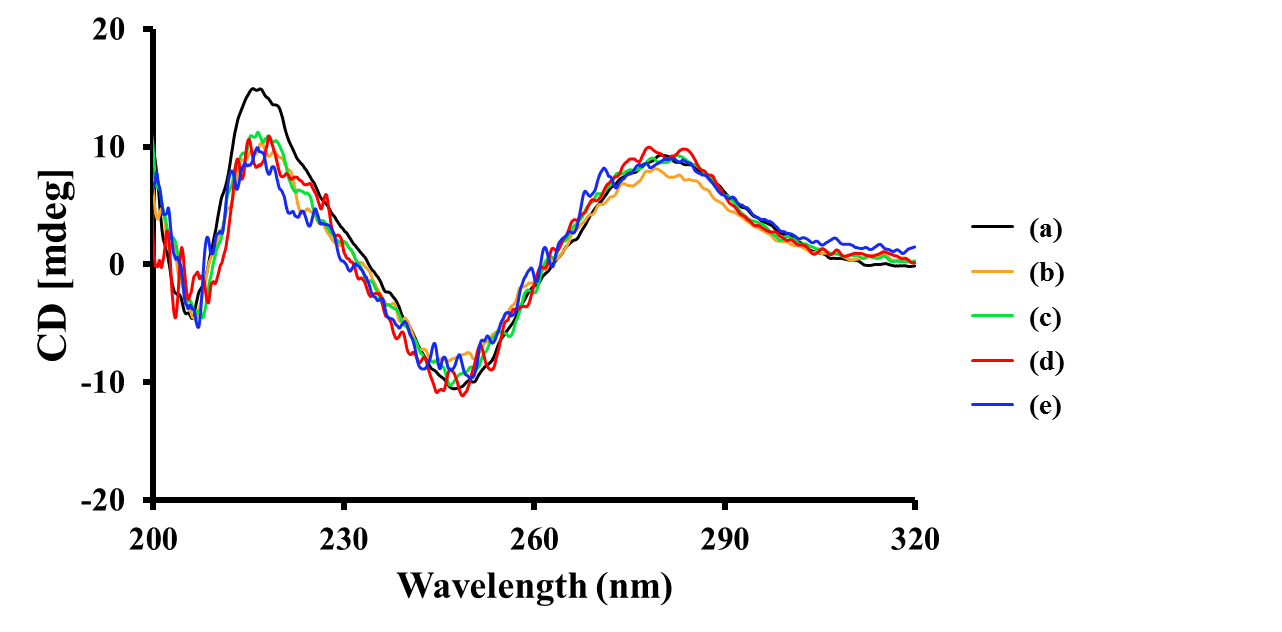


***Figure S8.*** Circular dichroism (CD) spectra of (a) Supra-TBA_15/29_ (8 μM), and (b−e) Supra-TBA_15/29_−GO (8 μM in term of Supra-TBA_15/29_) prepared at (b) 25, (c) 45, (d) 60, and (e) 90 ºC. The Supra-TBA_15/29_−GO was prepared from the Supra-TBA_15/29_ (2.5 μM) and GO (40 μg mL^−1^) and the obtained Supra-TBA_15/29_−GO was further purified and concentrated by centrifugation.

**
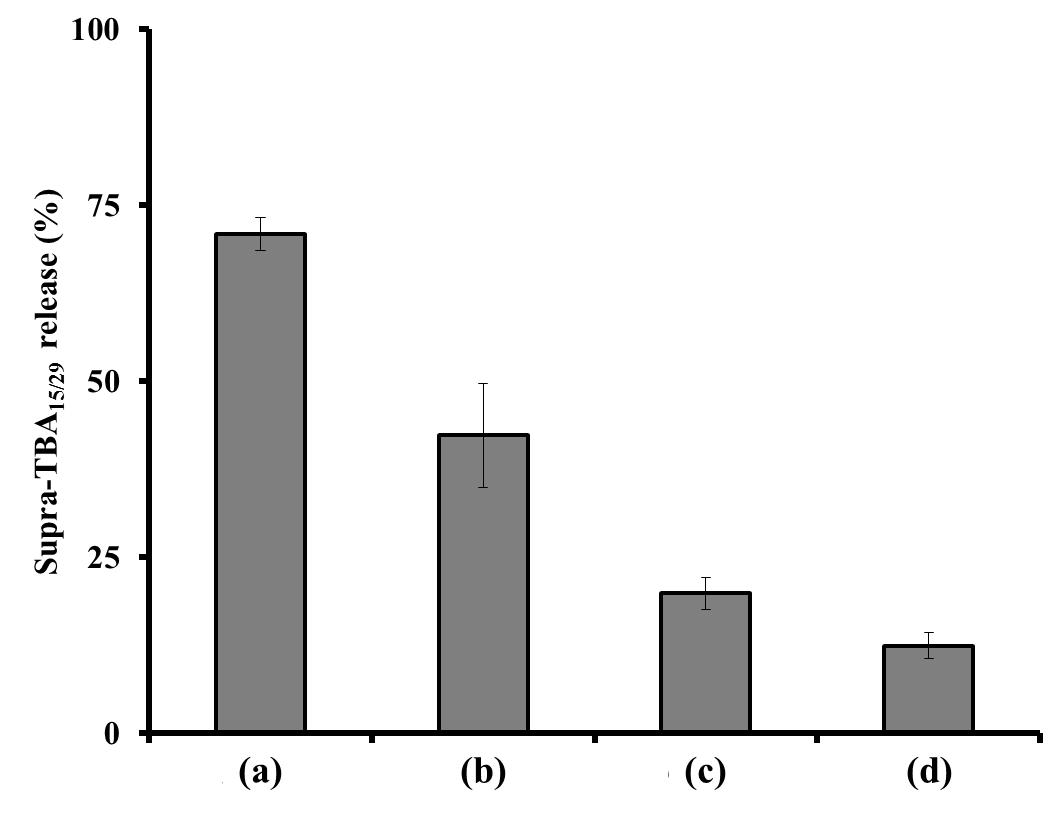
**

***Figure S9.*** Percentage of Supra-TBA_15/29_ released from Supra-TBA_15/29_−GO prepared at (a) 25, (b) 45, (c) 60, and (d) 90 ºC with the GO of 20 μg mL^−1^ after storing in plasma at 37 ºC for 2 h.


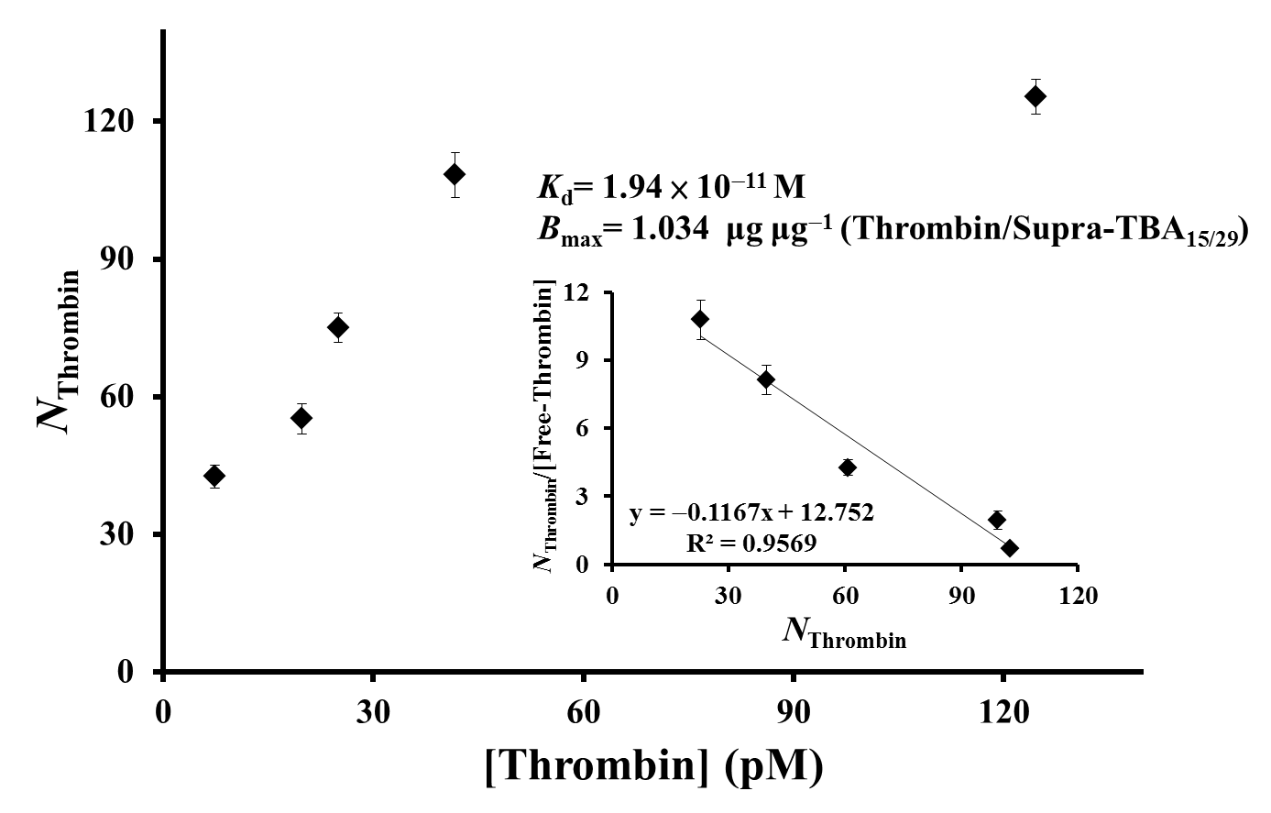


***Figure S10.*** Plot for calculating the dissociation constant *K*_d_ for thrombin and Supra-TBA_15/29_−GO. [*N*_Thrombin_] is the number of thrombin molecules bound to each Supra-TBA_15/29_ at equilibrium and [Free-Thrombin] is the free thrombin concentration at equilibrium. The error bars represent the standard deviations of experiments in triplicate.


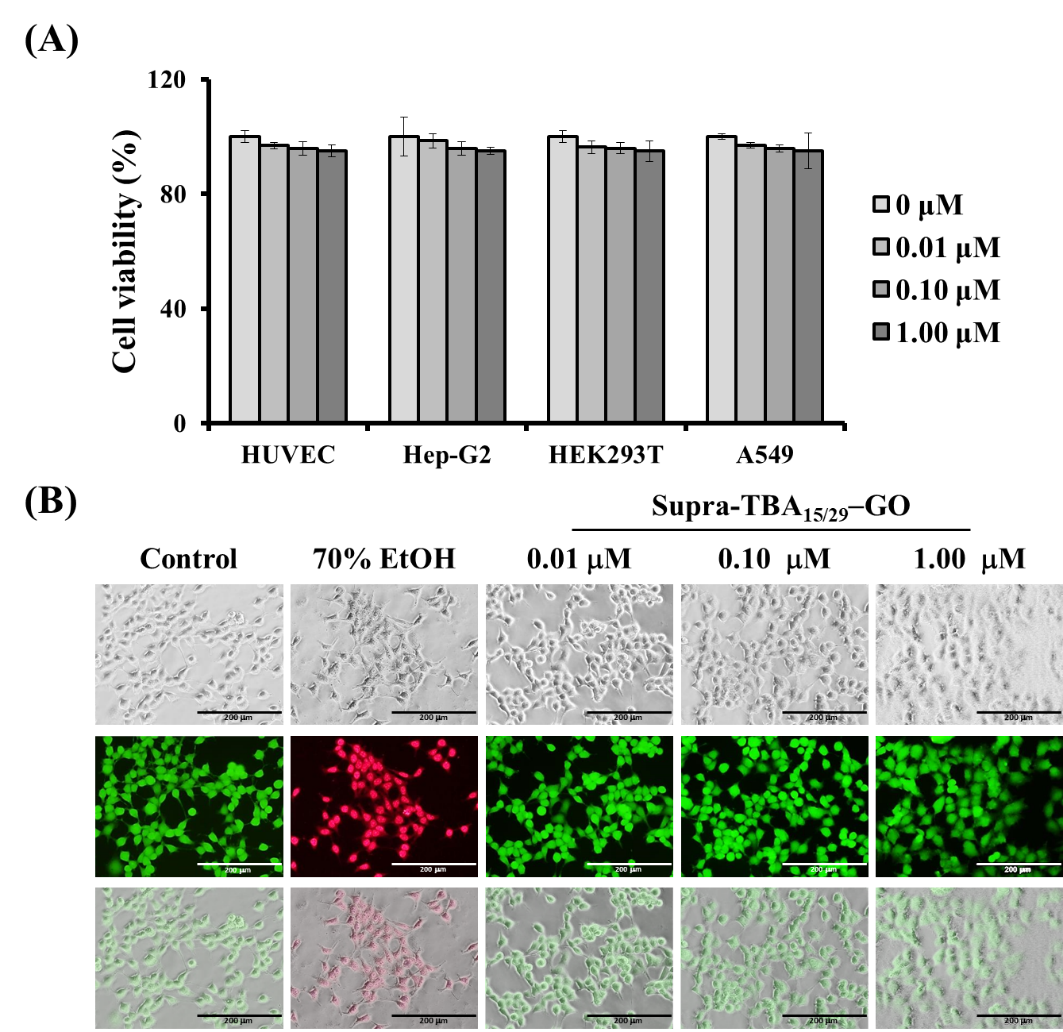


***Figure S11.*** The cytotoxicity of Supra-TBA_15/29_−GO toward different mammalian cells was assessed using the (A) MTT cell proliferation assay and (B) LIVE/DEAD assay. (A) Cell Viability of HUVEC, Hep-G2, HEK293T, and A549 cells incubated with Supra-TBA_15/29_−GO having different concentration (0−1.00 μM; in terms of TBA) in DMEM medium at 37 ºC with 5% CO_2_ atmosphere for 24 h. (B) Bright field, dark field and merged cell fluorescence images of HEK293T cells. Cells were also incubated in the same condition described as (A). Live cells produced green fluorescence and dead cells showed red fluorescence. LIVE/DEAD assay with PBS and 70% ethanol (EtOH) as negative control (NC) and positive control (PC), respectively. Error bars represent the standard deviation of three repeated measurements. Scale bar in (B) is 200 μm.

**
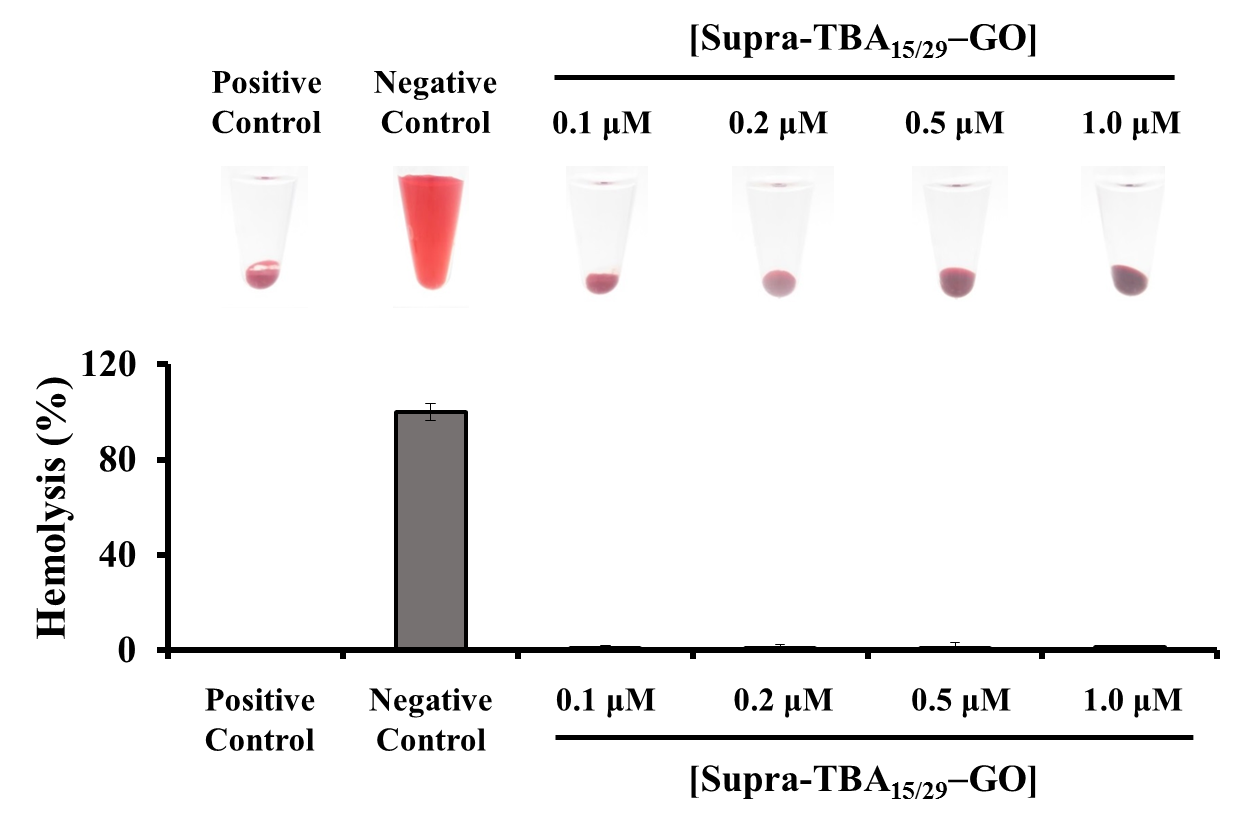
**

***Figure S12.*** Hemolysis assays for various concentrations of Supra-TBA_15/29_−GO (0.1−1.0 μM in terms of TBA) on RBCs. Hemolysis assays with PBS and DI water were used as negative control and positive control, respectively. Error bars represent the standard deviation of four repeated measurements.
